# Visceral adipose tissue area and proportion provide distinct reflections of cardiometabolic outcomes in weight loss; pooled analysis of MRI-assessed CENTRAL and DIRECT PLUS dietary randomized controlled trials

**DOI:** 10.1186/s12916-025-03891-9

**Published:** 2025-02-04

**Authors:** Hadar Klein, Hila Zelicha, Anat Yaskolka Meir, Ehud Rinott, Gal Tsaban, Alon Kaplan, Yoash Chassidim, Yftach Gepner, Matthias Blüher, Uta Ceglarek, Berend Isermann, Michael Stumvoll, Ilan Shelef, Lu Qi, Jun Li, Frank B. Hu, Meir J. Stampfer, Iris Shai

**Affiliations:** 1 The Health & Nutrition Innovative International Research Center, Department of Epidemiology, Biostatistics and Community Health Sciences, Faculty of Health Sciences, School of Public Health, Ben-Gurion University of the Negev, Beer-Sheva, 8410501 Israel; 2 Department of Engineering, Sapir Academic College, Shaar Hanegev, Israel; 3 Department of Health Promotion, School of Public Health, Faculty of Medicine and Sylvan Adams Sports Institute, Tel-Aviv University, Tel-Aviv, Israel; 4 Helmholtz Institute for Metabolic, Obesity and Vascular Research (HI-MAG) of the Helmholtz Zentrum München at the University of Leipzig and University Hospital Leipzig, Leipzig, Germany; 5 Institute of Laboratory Medicine, University of Leipzig Medical Center, Leipzig, Germany; 6 Department of Medicine, University of Leipzig, Leipzig, Germany; 7 Soroka University Medical Center, Beer-Sheva, Israel; 8 Department of Epidemiology, School of Public Health and Tropical Medicine, Tulane University, New Orleans, LA USA; 9 Department of Nutrition, Harvard T.H. Chan School of Public Health, Boston, MA USA; 10 Brigham and Women’s Hospital and Harvard Medical School, Boston, USA

**Keywords:** Visceral adipose tissue, Subcutaneous adipose tissue, Weight loss, Diabetes, Metabolic syndrome

## Abstract

**Background:**

Visceral adipose tissue (VAT) is well established as a pathogenic fat depot, whereas superficial subcutaneous adipose tissue (SAT) is associated with either an improved or neutral cardiovascular state. However, it is unclear to what extent VAT area (VATcm^2^) and its proportion of total abdominal adipose tissue (VAT%) are distinguished in predicting cardiometabolic status and clinical outcomes during weight loss.

**Methods:**

We integrated magnetic resonance imaging (MRI) measurements of VAT, deep-SAT, and superficial-SAT from two 18-month lifestyle weight loss clinical trials, CENTRAL and DIRECT PLUS (*n* = 572).

**Results:**

At baseline, the mean VATcm^2^ was 144.8cm^2^ and VAT% = 28.2%; over 18 months, participants lost 28cm^2^ VATcm^2^ (− 22.5%), and 1.3 VAT% units. Baseline VATcm^2^ and VAT% were similarly associated with metabolic syndrome, hypertension, and diabetes status, while VAT% better classified hypertriglyceridemia. Conversely, higher VATcm^2^ was associated with elevated high-sensitivity C-reactive protein (hsCRP), while VAT% was not. After 18 months of lifestyle intervention, both VATcm^2^ and VAT% loss were significantly associated with decreased triglycerides, HbA1c, ferritin, and liver enzymes, and increased HDL-c levels beyond weight loss (FDR < 0.05). Only VATcm^2^ loss was correlated with decreased HOMA-IR, chemerin, and leptin levels.

**Conclusions:**

MRI follow-up of 572 participants over 18 months of weight loss intervention suggests that although increased VATcm^2^ and VAT% exhibit similar clinical manifestations, it might be preferable to examine VAT% when exploring lipid status, while VATcm^2^ may better reflect inflammatory and glycemic states.

**Trial registration:**

CENTRAL (Clinical-trials-identifier: NCT01530724); DIRECT PLUS (Clinical-trials-identifier: NCT03020186).

**Supplementary Information:**

The online version contains supplementary material available at 10.1186/s12916-025-03891-9.

## Background

Although visceral adipose tissue (VAT) is widely recognized as a pathogenic fat depot, superficial subcutaneous adipose tissue (SAT) has been linked to improved indicators of cardiovascular health [1–4]. This contrast contributed to the controversy surrounding the distinction between the VAT area and its proportion of the total abdominal adipose tissue (%). While some reports explore metabolic complications of obesity with measures of VAT absolute area or volume [5–8], others refer to VAT proportion or other normalized indices of VAT as ratios that may better reflect cardiometabolic status and clinical outcomes [9–15]. Notably, some reports found VAT absolute quantity to be superior in predicting specific features independently (e.g., fasting insulin) and inferior in predicting others (e.g., fasting glucose) compared to the VAT ratio to SAT [16, 17].

This controversy is further increased by the impracticality of assessing abdominal fat depots in routine clinical settings with costly techniques such as dual-energy X-ray absorptiometry (DEXA), computed tomography (CT), and magnetic resonance imaging (MRI) [4, 18, 19]. To address this quantification challenge, several surrogate indices have been developed to serve as alternative estimators for assessing VAT quantity. Some of these indices are strictly anthropometric, such as the body mass index (BMI) and waist circumference (WC) [18, 20–22], and others incorporate additional characteristics such as demographic data (e.g., age, sex), plasma biomarkers (e.g., glucose, cholesterol, and amino acids) and bioelectrical impedance analysis (BIA) parameters [23–28]. However, compared to the numerous surrogate indices of VAT area, estimations of VAT proportion and changes in VAT area and proportion are lacking [4, 29].

In this study, we utilized pooled data from two 18-month lifestyle, randomized weight-loss clinical trials, CENTRAL and DIRECT PLUS, with 572 participants and MRI-assessed fat depots. We hypothesized that either the absolute area or proportion of VAT would better reflect obesity complications. To test this, we developed novel predictors to assess the VAT state and changes following lifestyle interventions.

## Methods

### Study design

This is a pooled analysis of two 18-month lifestyle intervention clinical trials, the CENTRAL (Clinical-trials-identifier: NCT01530724) and the DIRECT PLUS (Clinical-trials-identifier: NCT03020186) trials. Data from both studies were combined for the purposes of the present paper. The CENTRAL (*n* = 278, 2012–2014) and DIRECT PLUS (*n* = 294, 2017–2019) clinical trials were conducted in the same research center workplace in Dimona, Israel. The retention rates at 18 months were 86.3% and 89.8%, respectively, as previously described in detail [30, 31]. Accordingly, this pooled analysis included data on 572 participants at baseline and 528 participants who completed the trials. Of 572 participants from both trials, almost all MRI scans at baseline were eligible for quantification of the VAT area (*n* = 564; 99%) and VAT proportion (*n* = 553; 97%). Losses were due to technical reasons.

Both trials had similar inclusion criteria: WC > 102 cm for men and > 88 cm for women or dyslipidemia (serum triglycerides (TG) > 150 mg/dL and high-density-lipoprotein cholesterol (HDLc) < 40 mg/dL for men and < 50 mg/dL for women). DIRECT PLUS was limited to those over age 30. Both trials had nearly identical exclusion criteria, as fully described in Additional file 1: Methods S1. Both studies were approved and monitored by the Medical Ethics Board and Helsinki Committee of the Soroka University Medical Center. All participants provided written informed consent and received no financial compensation or gifts.

### Randomization and interventions

All diets aimed for moderate, long-term weight loss with restricted consumption of trans fats and refined carbohydrates and increased intake of vegetables. Lunch, commonly the primary meal in this population, was tailored to meet the specific dietary requirements of each group and was provided through the workplace cafeteria. All lifestyle education programs were provided to all groups by physicians, clinical dietitians, and fitness instructors at the same intensity. Randomization was performed with an equal allocation ratio across all treatment groups, stratified by sex and work site. The participants were aware of their assigned intervention (open-label). Study investigators assessing outcomes were blinded to the group assignments.

In the CENTRAL trial, the diet groups were low-fat or Mediterranean (MED)/low-carbohydrate. These groups were further divided after 6 months into groups with added physical activity (PA) or no added PA for the last 12 months of intervention. In the DIRECT PLUS trial, the diet groups included healthy dietary guidelines, MED, and green-MED, all of which were combined with PA. PA intensity was measured using metabolic equivalent for task (MET) units per week, in which each unit represents the ratio of work metabolic rate to resting metabolic rate [32]. The characteristics of each lifestyle intervention group are fully detailed in previous publications [30, 31].

### Outcome measures

Abdominal fat deposits were evaluated at baseline and 18 months later using 3-T MRI (Philips Ingenia 3.0 T). The scanner utilized a 3-dimensional modified DIXON imaging technique. A breath-hold technique was employed to prevent motion artifacts during abdominal scanning. The observers were blinded to the time point and treatment group in all quantifications and comparisons. Interclass and intraclass reliability were *r* > 0.96 (*p* < 0.001) [2, 30]. Perirenal fat was not considered visceral fat. Abdominal fat depots were quantified using MATLAB-based semiautomatic software [30, 31]. A continuous line was manually traced along the fascia superficialis to distinguish between the deep SAT and superficial SAT [2, 30]. Deep and superficial SAT were quantified exclusively in the abdominal region using MRI scans. These measurements do not account for total body SAT, including SAT located in the extremities. The scans included 2 axial slices, L4-L5 and L5-S1. Quantification of the fat mass regions included both the absolute area of each fat type and its proportion (percentage) of the total area of both fat types [VAT/(VAT + SAT)*100]. The entire protocol is reported in Additional file 1: Methods S2. Anthropometric parameters and blood biomarkers were measured at baseline and after 18 months of intervention (Additional file 1: Methods S3).

### Statistical analysis

A common coprimary outcome of both trials was VAT change following lifestyle interventions. This report presents continuous variables as the means (standard deviations) or medians (interquartile ranges), depending on the variables’ normal distribution. Nominal variables are expressed as numbers and percentages. Histograms of each variable were inspected to determine whether the variables were normally distributed. Nonnormal distributions were natural logarithm (ln) transformed. Changes in VAT, anthropometrics, and biomarkers were computed as changes relative to baseline [(time 18 − time 0)/time 0 × 100]. Differences between two groups of baseline characteristics were tested using the chi-square test for nominal variables and two sample *t*-test or Wilcoxon rank sum test for continuous variables. Sex-specific deciles of adipose tissue parameters were calculated per trial. Models were adjusted for trial to account for potential differences. The Kendall tau trend test was used to examine demographic and anthropometric measurements and blood biomarkers across adipose tissue deciles and groups of similar and opposite VAT areas and proportion medians via partial correlations. The ANCOVA test was also used to test demographic and anthropometric measurements and blood biomarkers across groups of similar and opposite VAT areas and proportion medians, with weight and deep SAT as covariates. Post hoc analyses were conducted using either an unpaired *t*-test or a Mann–Whitney *U* test, depending on the distribution of the variables, with Bonferroni correction applied for multiple comparisons. The correlations between VAT parameters were tested using Spearman’s correlation analysis. The metabolic syndrome criteria were assessed based on the harmonized criteria for clinical diagnosis of metabolic syndrome [33]. The Youden index was used to determine the optimal VAT cutoffs for metabolic morbidities [34]. We used generalized linear regression models (GLMs) to classify participants with metabolic abnormalities and evaluated their performance using the C-statistic, also known as the concordance statistic or the area under the receiver operating characteristic curve (AUC-ROC) [35]. We used the robust likelihood test to test whether a nonnested model fits better than a reference model. Least absolute shrinkage and selection operator (LASSO) regression was used to identify and evaluate predictors of VAT parameters at baseline and their changes following lifestyle intervention. Models were constructed for both sexes and for men only. The models were trained on 80% of the DIRECT PLUS data, tested on the remaining 20%, and validated on the CENTRAL data. Random splits of observations into training and testing sets were stratified by sex and VAT area or proportion quartiles, depending on the predicted VAT measure. Predictor variables included anthropometrics, demographics, and blood biomarkers available from both the CENTRAL and DIRECT PLUS trials (including glycemic and lipidic profiles, liver enzymes, inflammatory markers, and adipokines). The predictors were centered and scaled prior to model fitting. The LASSO penalty tuning parameter *λ* with the minimal average root mean square error (RMSE) across tenfold cross-validation and 10 repeats was chosen to compute the final model on the complete training data. All the statistical analyses were computed using R version 4.2.0, with the use of the following packages: “gtsummary,” “cutpointr,” “pROC”, “performance,” “nonnest2,” “caret,” “dplyr,” “ggplot2,” “corrplot” and “glmnet,” “interactions” [36–46].

## Results

### Baseline characteristics

The participants (Table 1) had a mean age of 49.5 ± 10.1 years, with an average BMI of 30.9 ± 3.9 kg/m^2^ and WC of 108.2 ± 9.7 cm (109.1 ± 9.1 cm for males and 101.4 ± 11.0 cm for females). Most participants were men (88.5%), 10.9% of participants had diabetes, and 62.6% had metabolic syndrome. The mean VAT area was 144.8 ± 58.5 cm^2^ and the mean VAT proportion was 28.2 ± 9.0%. Following the 18-month lifestyle intervention, participants lost − 2.6 ± 5.6 kg of body weight and − 4.8 ± 5.9 cm of their WC. The changes in visceral abdominal adipose depot parameters were − 27.9 cm^2^ (− 52.6 to − 6.4) for VAT area (− 22.5% (− 35.9 to − 5.7)) and − 1.3 ± 3.4% VAT proportion absolute units (− 3.8% (− 1.5 to 3.6)). The VAT/SAT ratio was 0.4 (0.3–0.5) and did not change following the intervention (Additional file 1: Table S1). At baseline, it exhibited a strong correlation with VAT proportion (*r* = 0.94, *p* < 0.001).

**Table 1:** Sex-specific baseline characteristics of the CENTRAL and DIRECT PLUS clinical trials participants

**Characteristic**^*1*^	***N***	**Overall**, *N* = 572^*2*^	**Male**, *N* = 506^*2*^	**Female**, *N* = 66^*2*^	***p* value**^*3*^	***q*-value**^*4*^
Age	572	49.5 ± 10.1	49.3 ± 10.1	51.1 ± 10.1	0.17	0.23
Weight, kg	572	92.6 ± 13.9	94.3 ± 13.1	79.4 ± 13.0	< 0.001	< 0.001
BMI, kg/m^2^	572	30.9 ± 3.9	30.9 ± 3.7	31.0 ± 5.2	0.87	0.87
Waist circumference, cm	571	108.2 ± 9.7	109.1 ± 9.1	101.4 ± 11.0	< 0.001	< 0.001
Diabetes	568	62 (10.9)	58 (11.5)	4 (6.2)	0.19	0.23
Metabolic syndrome	559	350 (62.6)	321 (65.0)	29 (44.6)	0.002	0.003
SSAT area, cm^2^	553	119.7 (91.5–162.9)	113.6 (87.9–151.9)	197.0 (159.0–256.2)	< 0.001	< 0.001
DSAT area, cm^2^	560	228.2 (179.9–290.2)	228.5 (178.5–293.9)	227.3 (187.4–268.4)	0.69	0.77
VAT area, cm^2^	564	134.8 (103.2–174.3)	139.3 (108.2–178.1)	105.4 (77.8–138.4)	< 0.001	< 0.001
VAT proportion, %	553	28.2 ± 9.0	29.3 ± 8.7	19.8 ± 6.7	< 0.001	< 0.001
VAT/SAT	553	0.4 (0.3–0.5)	0.4 (0.3–0.5)	0.2 (0.2–0.3)	< 0.001	< 0.001

^1^*BMI* Body mass index, *SSAT* Superficial subcutaneous adipose tissue, *DSAT* Deep subcutaneous adipose tissue, *VAT* Visceral adipose tissue, *SAT* Subcutaneous adipose tissue

^2^Values are presented as either the median (p25, p75) or the mean ± standard deviation for continuous variables, depending on their distribution, or as a number (%) for categorical variables

^3^Two sample *t*-test; Pearson’s chi-squared test; Wilcoxon rank sum test

^4^False discovery rate correction for multiple testing

Baseline VAT area and proportion sex-specific deciles showed parallel direct and significant correlation trends with age, blood pressure, and most blood biomarkers (FDR < 0.05) (Additional file 1: Table S2 and Fig. S1). However, VAT area and proportion exhibited dissimilar associations with anthropometric measurements and specific blood biomarkers. Specifically, VAT area was positively associated with WC (tau = 0.33, FDR < 0.001), chemerin (tau = 0.18, FDR < 0.001), high-sensitivity C reactive protein (hsCRP) (tau = 0.0.16, FDR < 0.001), alkaline phosphatase (ALKP), and alanine transaminase (ALT) (tau = 0.09, FDR = 0.04 for both), while the VAT proportion was not associated with these markers. Additionally, while VAT area demonstrated an increasing trend with body weight (tau = 0.21, FDR < 0.001) and leptin (tau = 0.19, FDR < 0.001), VAT proportion presented a decreasing trend with these measurements (tau = − 0.13 and − 0.12, FDR < 0.001).

**Fig. 1:**
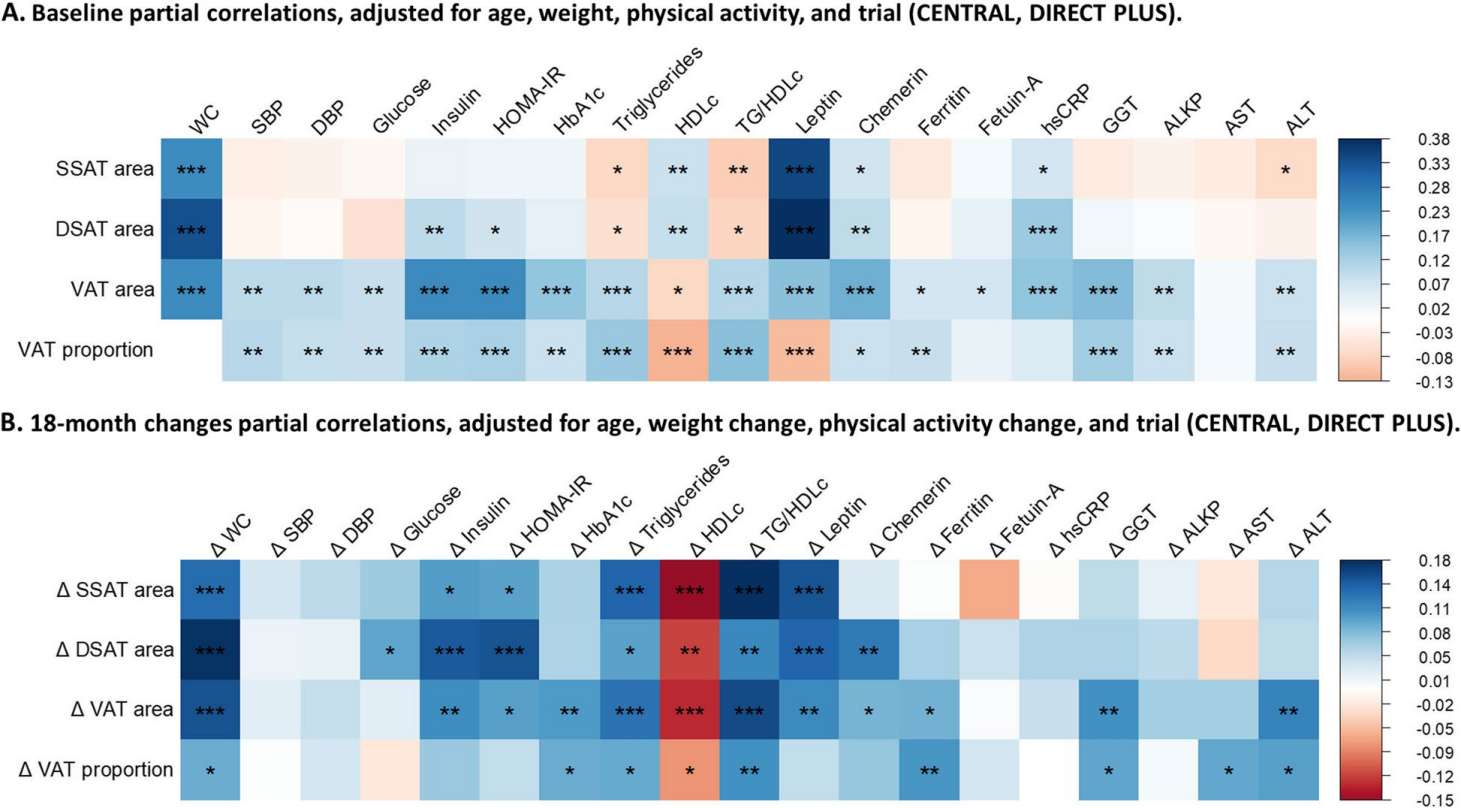
Abdominal adipose tissue area and proportion across various characteristics (baseline and 18-month changes). Heatmaps of Kendall’s tau partial correlations. **A** Baseline characteristics across abdominal adipose tissue sex-specific ranks, adjusted for age, weight, physical activity, and intervention trial (CENTRAL, DIRECT PLUS). SSAT area, *n* = 553; DSAT area, *n* = 560; VAT area, *n* = 564; VAT proportion, *n* = 533. **B** 18-month change characteristics across abdominal adipose tissue sex-specific ranks change, adjusted for age, weight loss, physical activity change, and intervention trial. Δ SAT area *n* = 430, Δ DSAT area *n* = 438, Δ VAT area *n* = 440, Δ VAT proportion *n* = 429. Correlations are color-coded with blue = positive correlation and red = negative correlation. Benjamini–Hochberg correction was used for multiple comparisons (FDR 5%). Asterisks (***, **, *) correspond to FDRs of 0.001, 0.01, and 0.05, respectively. Abbreviations: SSAT, superficial subcutaneous adipose tissue; DSAT, deep subcutaneous adipose tissue; VAT, visceral adipose tissue; WC, waist circumference; SBP, systolic blood pressure; DBP, diastolic blood pressure; HOMA-IR, homeostatic model assessment of insulin resistance; hsCRP, high-sensitivity C reactive protein; HDLc, high-density lipoprotein cholesterol; GGT, gamma-glutamyl transferase; ALKP, alkaline phosphatase; AST, aspartate transaminase; ALT, alanine transaminase

These trends remained following adjustment for age, weight, physical activity, and intervention trial (CENTRAL and DIRECT PLUS). In a partial correlation analysis adjusted for these covariates (Fig. 1A), all fat depot areas were directly and significantly associated with WC, leptin, chemerin, and hsCRP (FDR < 0.05). In contrast to VAT area, which was negatively associated with HDLc (tau = − 0.08, FDR < 0.05) and positively associated with TG and TG/HDLc (tau = 0.11, FDR < 0.001), superficial and deep SAT exhibited positive correlations with HDLc (tau = 0.08 and 0.09, FDR < 0.01) and inverse correlations with TG (tau = − 0.08 and − 0.06, FDR < 0.05) and TG/HDLc (tau = − 0.09 and − 0.08, FDR < 0.05). The VAT proportion association with TG and HDLc (tau = 0.14 and − 0.13, FDR < 0.001) appeared stronger than that of the VAT area (tau = 0.10 and − 0.08, FDR < 0.001). Unlike the VAT area, VAT proportion did not correlate with WC or hsCRP and was negatively correlated with leptin (FDR < 0.001).

### Baseline VAT area and proportion in relation to obesity complications

**Fig. 2:**
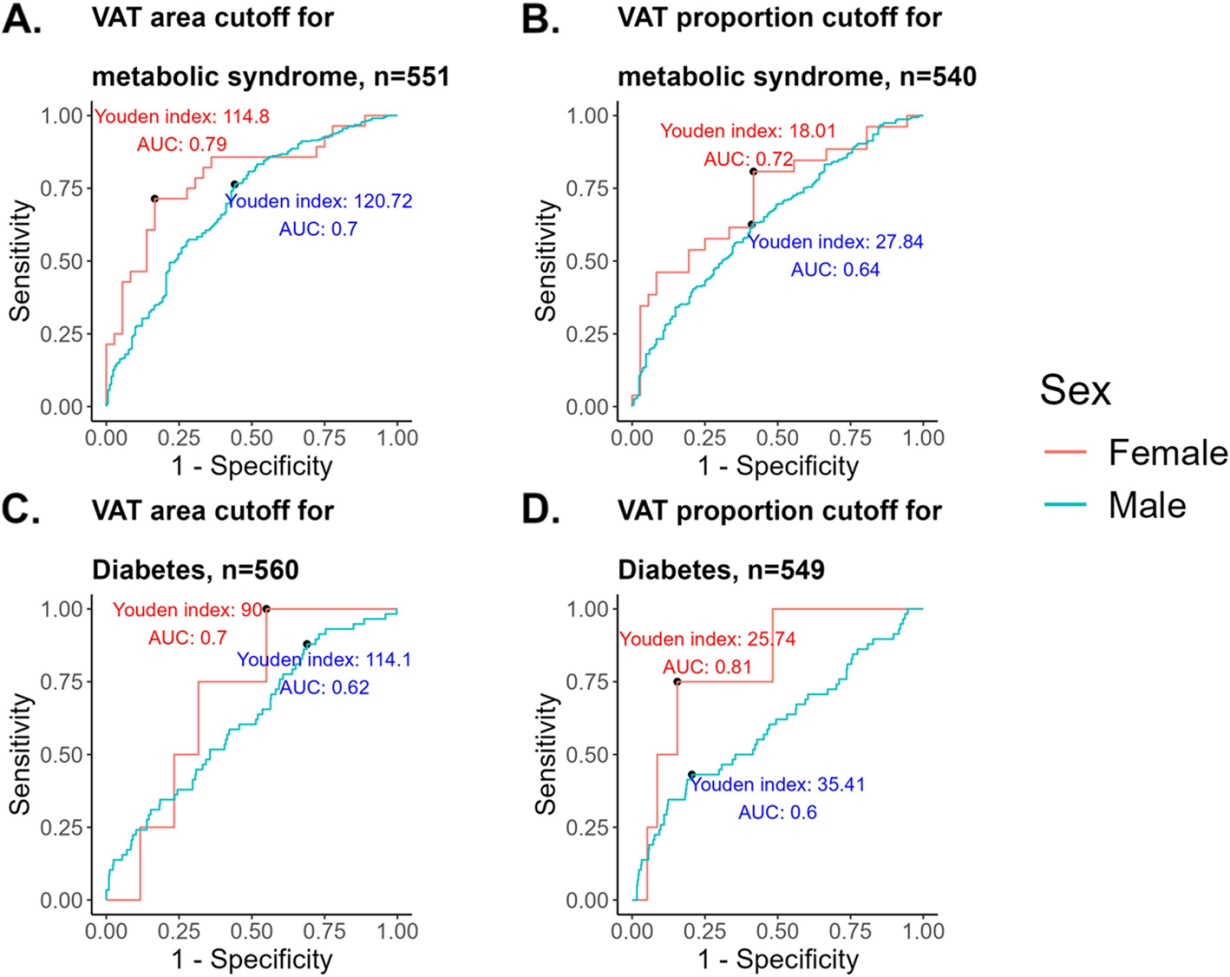
Sex-specific cutoff thresholds of visceral adipose tissue (VAT) area and proportion for metabolic morbidities. Receiver operating characteristic (ROC) curves that illustrate sex-specific cutoff thresholds for VAT area and VAT proportion in predicting metabolic syndrome and diabetes. The red line represents females, and the blue line represents males. VAT, visceral adipose tissue

Sex-specific cutoff values of VAT area and proportion were calculated for metabolic syndrome and diabetes status (Fig. 2, Additional file 1: Results S1).

**Fig. 3:**
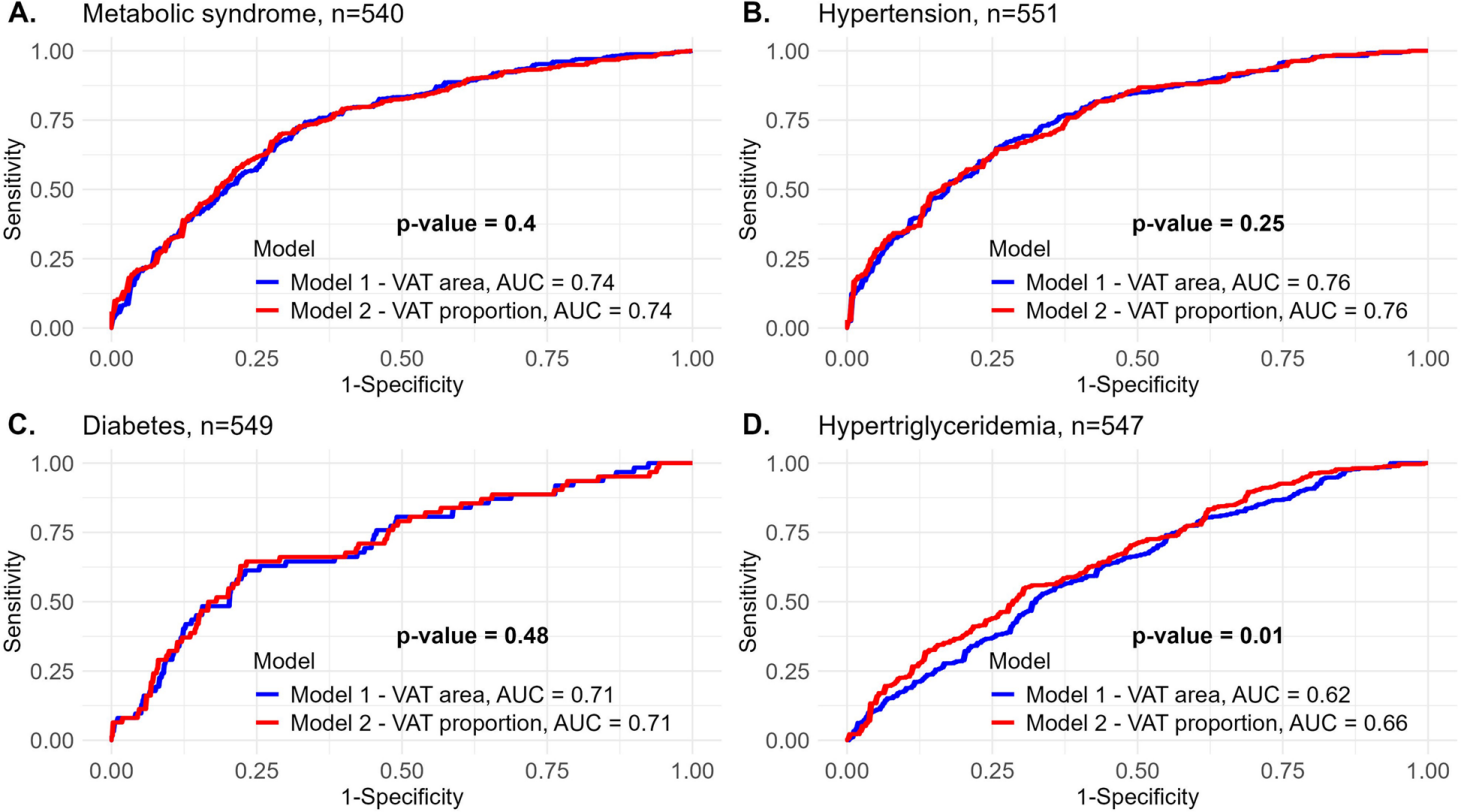
Visceral adipose tissue area and proportion predictions of obesity complications; receiver operating characteristic curve models. *n* = 540–551 participants. The ROC curves compare the performance of different logistic regression models in predicting the odds of obesity complications at baseline: metabolic syndrome (**A**), hypertension (**B**), diabetes (**C**), and hypertriglyceridemia (**D**). Two sets of models are evaluated for each morbidity state prediction: Model 1, represented by the blue curve, uses VAT area as a predictor variable, and Model 2, represented by the red curve, uses VAT proportion as a predictor variable. Both models are adjusted for trial type (CENTRAL and DIRECT PLUS), sex, age, and baseline weight

We further compared the VAT area and proportion prediction performances in classifying states of metabolic dysfunction at baseline, in adjustment for trial type, sex, age, and baseline weight (Fig. 3). VAT area and proportion seemed to similarly predict states of metabolic syndrome (AUC = 0.74 for both, *p* = 0.40), hypertension (AUC = 0.76 for both, *p* = 0.25), and diabetes (AUC = 0.71, *p* = 0.48). However, VAT proportion performed better at classifying participants with hypertriglyceridemia (AUC = 0.66) compared to VAT area (AUC = 0.62) (*p* = 0.01).

VAT area and proportion were correlated with each other (*r* = 0.68, *p* < 0.001). Nevertheless, distinct phenotypes of visceral adiposity could be classified for participants whose VAT area was above the median (men = 139 cm^2^, female = 105 cm^2^) and whose VAT proportion was below the median (men = 29%, female = 19%), and vice versa. Participants with higher VAT area and decreased VAT proportion had, by definition, higher SAT and increased weight. In multivariable analyses of groups with similar and opposite sex-specific VAT area and proportion medians, participants (13%) characterized by a top-median VAT area and low-median VAT proportion exhibited increased diastolic blood pressure, HbA1c, fasting insulin, HOMA-IR, ALT, AST, leptin, chemerin, and hsCRP, after controlling for weight and deep SAT, compared to the other groups (FDR < 0.05 for all) (Additional file 1: Tables S3 and S4). Alternatively, participants (14.1%) with low-median VAT areas and top-median VAT proportions presented similar adverse lipid profiles to those with higher VAT areas (Additional file 1: Table S4).

### Eighteen-month changes in abdominal adipose tissue depots

Despite the opposite associations at baseline between SAT and VAT regarding lipids profile, the loss of each fat compartment was associated with an improved lipids profile, even after adjustment for age, overall weight loss, and intervention trial (Fig. 1B). Changes in VAT area and proportion were both directly and significantly correlated with reduced WC, HbA1c, dyslipidemia, ferritin, GGT, and ALT (FDR < 0.05). However, some contrasts were noted; while VAT area loss was correlated with reduced insulin (tau = 0.11), HOMA-IR (tau = 0.10), leptin (tau = 0.11), and chemerin (tau = 0.08), FDR < 0.05 for all, VAT proportion loss was not (tau = 0.04–0.07, FDR = 0.09–0.28). Alternatively, VAT proportion loss was correlated with reduced AST (tau = 0.09, FDR = 0.05), while VAT area loss was not (tau = 0.06, FDR = 0.13).

### Prediction models of VAT baseline and 18-month change

Prediction models were developed for VAT area and proportion baseline and changes, utilizing either anthropometric measurements and demographic data, blood biomarkers, or a combination of both. Each model’s selected variables and performance metrics are presented in Additional file 1: Tables S5-S8.

**Fig. 4:**
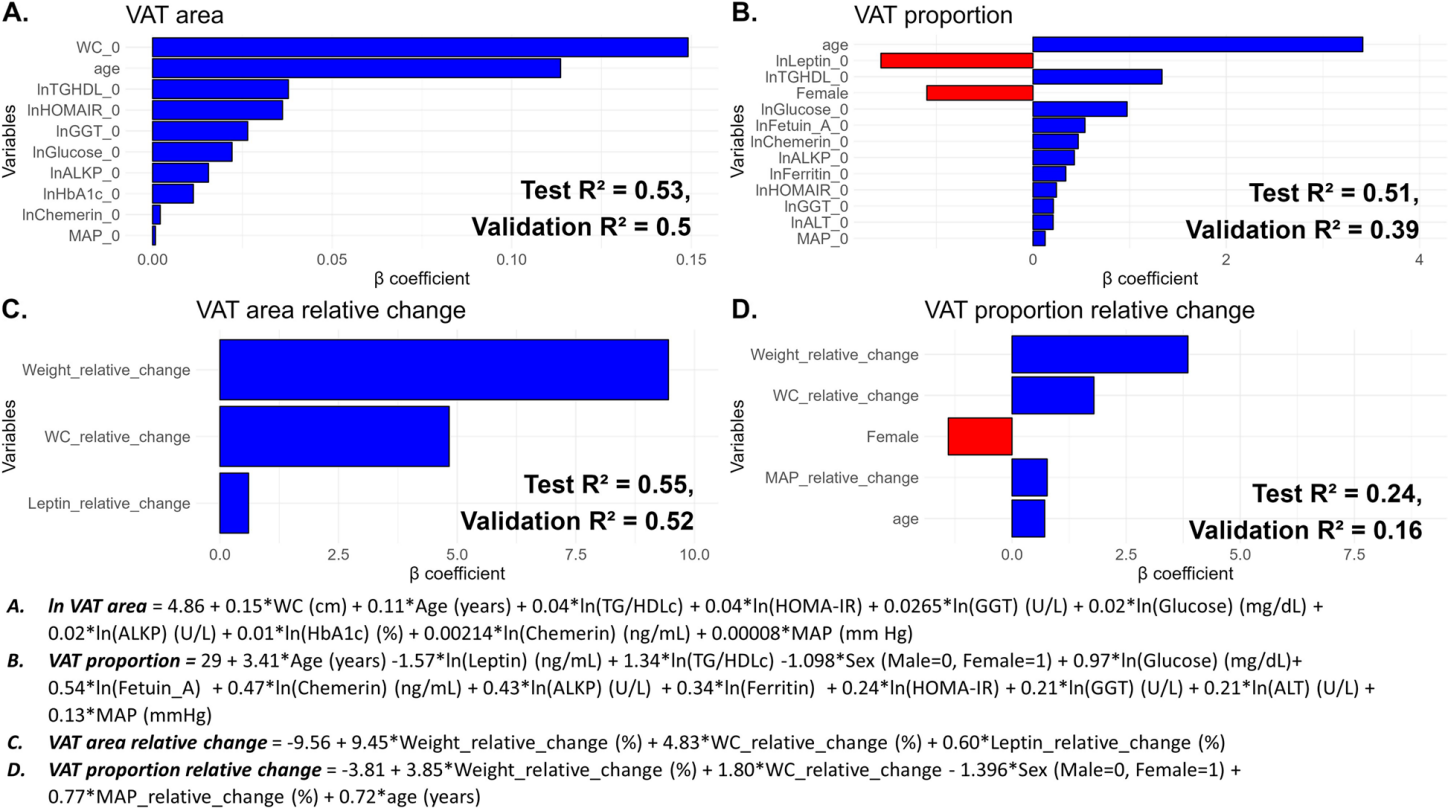
LASSO linear regression models of baseline and change VAT area and proportion. The *x*-axis displays the variables selected by the LASSO model, and the *y*-axis represents the estimated β-unstandardized coefficients. The magnitude and direction by which each variable affects the baseline VAT area (**A**) and proportion (**B**) and the 18-month relative changes in VAT area (**C**) and proportion (**D**) are represented by the color (blue for positive associations and red for negative associations) and length of the bars. Baseline VAT area (**A**) model was trained on a set of 227 participants, tested on 55 participants and validated on 259 participants. Baseline VAT proportion (**B**) model was trained on a set of *n* = 218 participants, tested on *n* = 53 participants and validated on *n* = 143 participants. The VAT area change (**C**) model was trained on a set of *n* = 180 participants, tested on *n* = 46 participants and validated on *n* = 207 participants. The VAT proportion change (**D**) model was trained on a set of 172 participants, tested on 43 participants and validated on 212 participants. Abbreviations: LASSO, least absolute shrinkage and selection operator; VAT, visceral adipose tissue; WC, waist circumference; MAP, mean arterial pressure; TG, triglycerides; HDLc, high-density lipoprotein cholesterol; HOMA-IR, homeostatic model assessment of insulin resistance; GGT, gamma-glutamyl transferase; ALKP, alkaline phosphatase; hsCRP, high-sensitivity c-reactive protein; ALT, alanine transaminase

The best-performing prediction model for baseline VAT area included a combination of anthropometrics, demographics, and blood biomarkers (Additional file 1: Table S5). It was trained on data from 227 DIRECT PLUS participants, tested on 55, and validated on 259 CENTRAL participants, with similar characteristics across the datasets (Additional file 1: Table S9). The cross-validation models had an RMSE of 0.27 and *R*^2^ of 0.44. The final model applied to the testing and validation datasets had RMSEs of 0.26 and 0.40 and *R*^2^ of 0.53 and 0.50, respectively. Selected predictors included WC, MAP, age, TG/HDLc, HbA1c, HOMA-IR, glucose, GGT, ALKP, and chemerin (Fig. 4A).

Similarly, the best-performing model for estimating baseline VAT proportion included similar predictors such as age and lipidic and glycemic indicators but did not include any anthropometric measurements. Additional selected variables were sex, ALT, fetuin-A, ferritin, and leptin (Fig. 4B, Additional file 1: Table S6). The model was trained on *n* = 218 participants (RMSE = 6.87, *R*^2^ = 0.37), tested on *n* = 53 participants (RMSE = 6.55, *R*^2^ = 0.51), and validated on *n* = 142 participants (RMSE = 6.7, *R*^2^ = 0.39).

As for VAT area change, the best predictor for testing data included only anthropometric measurements (RMSE = 15.33, *R*^2^ = 0.59), while the validation data model included both anthropometrics and blood biomarkers (RMSE = 52.1, *R*^2^ = 0.52), focusing on changes in weight, WC, MAP, and leptin (Fig. 4C, Additional file 1: Table S7).

The best model for predicting VAT proportion change used only anthropometric and demographic data. The model was trained on data from 172 participants (RMSE = 11.32, *R*^2^ = 0.15), tested on data from 43 participants (RMSE = 11.80, *R*^2^ = 0.24), and validated on data from 212 participants (RMSE = 18.72, *R*^2^ = 0.16) (Fig. 4D, Additional file 1: Table S8). Due to limited data, sex-specific predictors were only developed for males (Additional file 1: Fig. S2). Additional models were developed to account for the contribution of PA on VAT area and proportion and their change over time (Additional file 1: Figs. S3 and S4). An increase in MET/week predicted a higher loss of both VAT area and proportion.

For clinical practice applications, additional models were developed for both men and women, as well as for men only, that strictly utilize biomarkers commonly employed in clinical settings (Additional file 1: Figs. S5 and S6).

## Discussion

This pooled analysis of two 18-month lifestyle randomized controlled trials (*n* = 572) revealed notable differences in the parameters of abdominal VAT distribution. VAT area and VAT proportion are associated with similar metabolic indicators, with higher values corresponding to a worsened cardiometabolic state. However, VAT proportion was more strongly associated with lipid status, whereas VAT area was more strongly linked to glucose metabolism and inflammation biomarkers.

Several limitations should be acknowledged. First, although multislice volume imaging is considered the gold standard for measuring adipose tissue, it is achieved through a CT scan that involves radiation exposure [47]. Consequently, we opted for the safer MRI option, even though it measured abdominal adipose tissue area rather than volume. However, we calculated these areas as the means of two images at the L4-L5 and L5-S1 intervertebral spaces. Additionally, we observed high inter- and intraclass correlations (*r* > 0.96; *p* < 0.001), supporting their reproducibility. Second, the trials were conducted in a workplace environment with a predominantly male workforce, leading to 88.5% of the participants being men. Hence, we identified predictors for both sexes, accounting for sex, and for men only, but not for women only. Third, total fat mass was not assessed in our MRI measurements, limiting our ability to quantify VAT as a proportion of total body fat or to draw conclusions about total body SAT. Therefore, our discussion focuses on VAT as a proportion of total abdominal fat and on abdominal SAT. Future studies should investigate the roles of VAT relative to total fat mass and extremity SAT in cardiometabolic health. The strengths of the analysis include its large sample size for men and high retention rates within two relatively large and long clinical trials conducted in the same workplace for the same duration with similar inclusion and exclusion criteria. Furthermore, both trials measured VAT parameters using the same 3-T MRI analysis.

The VAT area and proportion were closely correlated and similarly associated with various cardiometabolic biomarkers, including hypertension, impaired glycemic and lipidic profiles, liver dysfunction, and elevated chemerin. However, while both VAT parameters were positively correlated with TG and negatively correlated with HDLc, SAT had opposite associations with these biomarkers. These findings are in accordance with the well-established association of superficial SAT with improved indicators of cardiovascular health [1–3]. This may explain why VAT proportion demonstrated a stronger correlation with TG and HDLc compared to VAT area and was a superior predictor of hypertriglyceridemia (*p* = 0.01).

More discrepancies have been noted, with body weight and WC presenting different trends across VAT area and proportion. While VAT area was positively correlated with weight and WC, VAT proportion was inversely related to weight and had no significant association with WC. These findings are attributed to the stronger association of weight and WC with SAT rather than with VAT and are consistent with previous reports that found VAT area to be greater in patients with obesity than in patients without obesity, in contrast to VAT proportion, which was similar in these groups [9].

The heterogeneous phenotypes of visceral obesity were classified to further explore the associations of VAT area and proportion with adverse health indicators. Naturally, participants presenting both increased (above median) VAT and SAT areas (i.e., low VAT proportion) were characterized by higher SAT, WC, and weight. As deep SAT was found to be independently associated with increased insulin resistance [30], we performed multivariable analyses between the visceral adiposity phenotype groups, controlling for weight and deep SAT. Participants with a high VAT area and low VAT proportion (*n* = 72) presented a worsened metabolic state compared to those with a low VAT area and high VAT proportion (*n* = 78). Specifically, they had higher insulin resistance and increased HbA1c levels (FDR < 0.01). This finding is in accordance with previous reports that VAT area is superior to VAT/SAT for predicting fasting insulin [16]. However, the diverse visceral adiposity phenotypes presented similar adverse lipid profiles, repeatedly revealing the strong association of increased VAT proportion with a poor lipidic state, even in the presence of a relatively low VAT area.

These findings may reflect the distinct metabolic properties of VAT and SAT adipocytes. VAT adipocytes exhibit higher metabolic activity and greater sensitivity to lipolysis compared to SAT adipocytes, resulting in increased free fatty acid (FFA) release and subsequent elevated very low-density lipoprotein (VLDL) secretion by the liver [48]. This heightened lipolytic activity may explain the stronger association of VAT proportion with lipid profiles compared to VAT area alone.

Conversely, the stronger link between VAT area and inflammatory and glycemic profiles could align with the “portal theory.” This theory suggests that VAT, primarily drained by the portal vein, directly delivers high concentrations of FFAs and pro-inflammatory factors to the liver, impairing hepatic metabolism and promoting insulin resistance [49, 50]. Therefore, the absolute VAT volume may better capture the magnitude of metabolites delivered to the liver, providing a more accurate reflection of inflammatory and glycemic states compared to VAT proportion.

While these interpretations provide plausible mechanisms, they remain speculative. Further studies are needed to unravel the complex interplay between adipose tissue depots, liver function, and systemic metabolism to clarify these relationships.

After 18 months, changes in weight and WC were modest, averaging − 2.6 ± 5.6 kg and − 4.8 ± 5.9 cm, respectively. Notably, previous evidence indicated that each kilogram of weight loss corresponds to a 3–4 cm^2^ reduction in VAT, while a 1-cm decrease in WC is associated with an approximate 5 cm^2^ (~ 4%) reduction in VAT [51]. Participants also increased their PA during the trials (+ 7.3 MET/week (Table S1)). Evidence further indicates that PA alone can independently reduce VAT by approximately 6%, even in the absence of weight loss [52]. These findings emphasize that, despite modest weight loss, reductions in abdominal adipose depots provided meaningful insights into improved cardiometabolic health outcomes.

Although SAT was beneficially associated with lipid profiles at baseline, its reduction was correlated with further improvements in these markers. Additionally, WC, which was not associated with VAT proportion at baseline, became a significant correlate of its change, likely due to VAT’s greater sensitivity to weight reduction [53, 54]. Variables selected for LASSO prediction formulas reflected the distinct associations of VAT area and proportion with anthropometric, demographic, and blood biomarkers measurements at baseline and of their changes. Specifically, older age was highly predictive of both increased VAT area and proportion, in agreement with other reports [7, 53, 55]. However, WC was predictive of VAT area, but not VAT proportion. Alternatively, male sex and lower levels of leptin were predictive of a higher VAT proportion but not of its area [4, 53, 55, 56]. The latter is explained by leptin’s higher secretion rates in SAT than in VAT [57]. Although indicators of poor lipid and glycemic profiles, along with elevated levels of liver enzymes and several adipokines, were important predictors of increased baseline VAT area and proportion, both of their changes were mainly predicted by a combination of anthropometric measurements.

## Conclusions

Although VAT area and proportion are highly correlated, each parameter holds distinct attributes of cardiometabolic state. While the VAT proportion is more strongly associated with a poor lipid state, the VAT area better reflects the inflammatory state and glycemic profile during weight loss. In contrast, superficial and deep SAT depots demonstrated favorable associations with lipid profiles, highlighting their potential cardioprotective role. These findings indicate the complexity of VAT dynamics and emphasize the relevance of personalized approaches in targeting visceral adiposity for cardiometabolic health improvement.

## Supplementary Information

Additional File 1: Methods S1: Exclusion criteria of the CENTRAL and DIRECT PLUS trials. Methods S2: Magnetic resonance imaging. Methods S3: Clinical parameters and laboratory methodology. Results S1: Cutoff values for metabolic syndrome and diabetes at baseline. Table S1: Baseline, end of intervention, and changes in key metabolic and adiposity variables. Table S2: Baseline characteristics of the CENTRAL and DIRECT PLUS clinical trials participants across sex-specific deciles of visceral adipose tissue proportion. *n* = 572 participants. Fig. S1: Baseline visceral abdominal adipose tissue area and proportion across various characteristics; Heatmap of Kendall’s tau correlations. Table S3: Visceral adiposity phenotypes of similar and opposite sex-specific visceral adipose tissue area and proportion medians. *N* = 553. Table S4: Post hoc analysis of visceral adiposity phenotypes of similar and opposite sex-specific visceral adipose tissue area and proportion medians. *N* = 553. Table S5: LASSO developed models for baseline VAT area. Table S6: LASSO developed models for baseline VAT proportion. Table S7: LASSO developed models for VAT area relative change. Table S8: LASSO developed models for VAT proportion relative change. Table S9: Participants’ characteristics in the training and testing data sets for baseline VAT area model development. Fig. S2: LASSO linear regression models of baseline and change VAT area and proportion (men only). Fig. S3: LASSO linear regression models of baseline and change VAT area and proportion while accounting for physical activity. Fig. S4: LASSO linear regression models of baseline and change VAT area and proportion while accounting for physical activity (men only). Fig. S5: LASSO linear regression models of baseline and change VAT area and proportion for clinical practice application. Fig. S6: LASSO linear regression models of baseline and change VAT area and proportion for clinical practice application (men only).

## Data Availability

The majority of results corresponding to the current studies are included in the article or uploaded as supplementary material. No further data are available.

## Abbreviations

ALKP: Alkaline phosphatase
ALT: Alanine transaminase
AST: Aspartate transaminase
BMI: Body mass index
CT: Computed tomography
DBP: Diastolic blood pressure
DEXA: Dual energy x-ray absorptiometry
FDR: False discovery rate
FFA: Free fatty acid
GGT: Gamma-glutamyl transferase
HDG: Healthy dietary guidelines
HDLc: High-density lipoprotein cholesterol
HOMA-IR: Homeostatic model assessment of insulin resistance
hsCRP: High-sensitivity C-reactive protein
LASSO: Least absolute shrinkage and selection operator
MAP: Mean arterial pressure
MED: Mediterranean
MET: Metabolic equivalent for task
MRI: Magnetic resonance imaging
PA: Physical activity
SAT: Subcutaneous adipose tissue
SBP: Systolic blood pressure
TG: Triglycerides
VAT: Visceral adipose tissue
VLDL: Very low-density lipoprotein
WC: Waist circumference
