# Correction: Visceral adipose tissue area and proportion provide distinct reflections of cardiometabolic outcomes in weight loss; pooled analysis of MRI-assessed CENTRAL and DIRECT PLUS dietary randomized controlled trials

**DOI:** 10.1186/s12916-025-03939-w

**Published:** 2025-02-18

**Authors:** Hadar Klein, Hila Zelicha, Anat Yaskolka Meir, Ehud Rinott, Gal Tsaban, Alon Kaplan, Yoash Chassidim, Yftach Gepner, Matthias Blüher, Uta Ceglarek, Berend Isermann, Michael Stumvoll, Ilan Shelef, Lu Qi, Jun Li, Frank B. Hu, Meir J. Stampfer, Iris Shai

**Affiliations:** 1 The Health & Nutrition Innovative International Research Center, Department of Epidemiology, Biostatistics and Community Health Sciences, Faculty of Health Sciences, School of Public Health, Ben-Gurion University of the Negev, Beer-Sheva, 8410501 Israel; 2 Department of Engineering, Sapir Academic College, Shaar Hanegev, Israel; 3 Department of Health Promotion, School of Public Health, Faculty of Medicine and Sylvan Adams Sports Institute, Tel-Aviv University, Tel-Aviv, Israel; 4 Helmholtz Institute for Metabolic, Obesity and Vascular Research (HI-MAG) of the Helmholtz Zentrum München at the University of Leipzig and University Hospital Leipzig, Leipzig, Germany; 5 Institute of Laboratory Medicine, University of Leipzig Medical Center, Leipzig, Germany; 6 Department of Medicine, University of Leipzig, Leipzig, Germany; 7 Soroka University Medical Center, Beer-Sheva, Israel; 8 Department of Epidemiology, School of Public Health and Tropical Medicine, Tulane University, New Orleans, LA USA; 9 Department of Nutrition, Harvard T.H. Chan School of Public Health, Boston, MA USA; 10 Brigham and Women’s Hospital and Harvard Medical School, Boston, USA

**Correction: BMC Med 23, 57 (2025)**

**https://doi.org/10.1186/s12916-025-03891-9**

Following the publication of the original article [1], the authors wish to note that in the sentence, '[…] high-sensitivity C-reactive protein (hsCRP) (tau = 0.0.16, FDR < 0.001)' in the Results section, '0.0.16' should instead read '0.16'.
