# Supplementary material for: Visceral adipose tissue area and proportion provide distinct reflections of cardiometabolic outcomes in weight loss; pooled analysis of MRI-assessed CENTRAL and DIRECT PLUS dietary randomized controlled trials
Source: BMC Med. 2025 Feb 4;23:57. doi: 10.1186/s12916-025-03891-9 (PMC11792534; doi:10.1186/s12916-025-03891-9)
Supplement: Supplementary file 1 — Additional File 1: Methods S1: Exclusion criteria of the CENTRAL and DIRECT PLUS trials. Methods S2: Magnetic resonance imaging. Methods S3: Clinical parameters and laboratory methodology. Results S1: Cutoff values for metabolic syndrome and diabetes at baseline. Table S1: Baseline, end of intervention, and changes in key metabolic and adiposity variables. Table S2: Baseline characteristics of the CENTRAL and DIRECT PLUS clinical trials participants across sex-specific deciles of visceral adipose tissue proportion. n = 572 participants. Fig. S1: Baseline visceral abdominal adipose tissue area and proportion across various characteristics; Heatmap of Kendall’s tau correlations. Table S3: Visceral adiposity phenotypes of similar and opposite sex-specific visceral adipose tissue area and proportion medians. N = 553. Table S4: Post hoc analysis of visceral adiposity phenotypes of similar and opposite sex-specific visceral adipose tissue area and proportion medians. N = 553. Table S5: LASSO developed models for baseline VAT area. Table S6: LASSO developed models for baseline VAT proportion. Table S7: LASSO developed models for VAT area relative change. Table S8: LASSO developed models for VAT proportion relative change. Table S9: Participants’ characteristics in the training and testing data sets for baseline VAT area model development. Fig. S2: LASSO linear regression models of baseline and change VAT area and proportion (men only). Fig. S3: LASSO linear regression models of baseline and change VAT area and proportion while accounting for physical activity. Fig. S4: LASSO linear regression models of baseline and change VAT area and proportion while accounting for physical activity (men only). Fig. S5: LASSO linear regression models of baseline and change VAT area and proportion for clinical practice application. Fig. S6: LASSO linear regression models of baseline and change VAT area and proportion for clinical practice application (men only). [file 12916_2025_3891_MOESM1_ESM.docx]

Supplemental Material

**Methods S1. Exclusion criteria of CENTRAL and DIRECT PLUS trials.**

Exclusion criteria for both trials were serum creatinine level≥2mg/dL, disturbed liver function, inability to partake in physical activity (PA), participation in a different trial, pregnancy or lactation, a significant illness that might require hospitalization, active cancer or chemotherapy treatment at present or three years before the trial. The CENTRAL trial also excluded subjects who were physically active (>3 hours per week) or had high triglycerides (>400 mg/dL). The DIRECT PLUS trial further excluded subjects who were treated with Warfarin (given its interaction with vitamin K) or had a pacemaker or platinum implantation.

**Methods S2. Magnetic resonance imaging.**

To assess body fat depots, 45-minute 3-Tesla magnetic resonance imaging (MRI) scans (Ingenia 3.0 T, Philips Healthcare) were performed at baseline and after 18 months in both trials. The scanner utilized a 3-dimensional modified DIXON imaging technique without gaps (2 mm thickness and 2 mm of spacing) and a fast-low-angle shot sequence with a multiecho 2-excitation pulse sequence for phase-sensitive encoding of fat and water signals (repetition time, 3.6 ms; echo time 1, 1.19 ms; echo time 2, 2.3 ms; FOV 520×440×80 mm; 2×1.4×1 mm voxel size). Four images of the phantoms were generated, including in-phase, out-phase, fat phase, and water phase [1]. A breath-hold technique was used to avoid motion artifacts when the abdomen was scanned. In all of the quantifications and comparisons, observers were blinded to time point and group treatment. All fat depots were assessed by 1 or 2 raters. Interclass and intraclass reliability were r > 0.96 (p < 0.001) [2, 3]. Abdominal fat was quantified using MATLAB-based semiautomatic software. A continuous line was manually drawn over the fascia superficialis to differentiate between the deep SAT and superficial SAT [2, 3]. Scans included 2 axial slices, L4-L5 and L5-S1. Quantification of the fat mass regions included both the absolute area of each fat type and its proportion (percentage) of the total area of all fat types.

**Methods S3. Clinical parameters and laboratory methodology.**

Measurements were taken at baseline and after 18-months of intervention. Height was measured to the nearest millimeter using a standard wall-mounted stadiometer. Bodyweight was measured without shoes to the nearest 0.1 kg. Waist circumference (WC) was measured halfway between the last rib and the iliac crest to the nearest millimeter by standard procedures using an anthropometric measuring tape. Two blood pressure (BP) measurements were recorded after resting using an automatic BP monitor (Accutorr-4, Datascope); the mean of the two was calculated.

Laboratory methodology:

Blood samples were obtained at 8:00 AM after a 12-hour fast and were subsequently centrifuged and stored at -80°C. Serum total cholesterol (TC; coefficient-of-variation (CV), 1.3%), HDL-c, low-density-lipoprotein-cholesterol (LDL-c), and TG (CV, 2.1%) were determined enzymatically with a Cobas 8000 automatic analyzer (Roche). Plasma levels of high-sensitivity C-reactive protein (hsCRP) were measured by a Tina-quant® hsCRP assay from Roche. Plasma glucose levels were measured by Roche GLUC3 (hexokinase method). Plasma insulin levels were measured with an enzyme immunometric assay (immulite automated analyzer, Diagnostic Products, coefficient of variation [CV], 2.5%) in CENTRAL trial and with a Roche Elecsis assay in DIRECT PLUS trial. The homeostatic model of insulin resistance (HOMA-IR) was calculated as follows: insulin (μIU/ml)×glucose (mg/dl)/405) [4]. Plasma leptin levels were assessed by ELISA (Mediagnost, CV, 2.4%). All biochemical analyses were performed at the University of Leipzig, Germany.

**Results S1. Cutoff values for metabolic syndrome and diabetes at baseline.**

The cutoff values for metabolic syndrome at baseline were 120.72 cm^2^ VAT area (AUC=0.70) and 27.84% VAT proportion (AUC=0.64) for men and 114.8 cm^2^ VAT area (AUC=0.79) and 18.01% VAT proportion for women (AUC=0.72). The cutoff values for diabetes status at baseline were 114.10 cm^2^ VAT area (AUC=0.62) and 35.41% VAT proportion (AUC=0.60) for men and 90 cm^2^ VAT area (AUC=0.70) and 25.74% VAT proportion for women (AUC=0.81).

**Table S1. Baseline, end of intervention, and change in key metabolic and adiposity variables.**

| **Characteristic** | **Baseline** | **End of Intervention** | **Change** |
| --- | --- | --- | --- |
| **Abdominal adipose tissue depots parameters** | | | |
| **VAT area, cm²** | 134.8 (103.2 – 174.3) | 103.7 (78.2 – 130.8) | -27.9 (-52.6 – -6.4) |
| **DSAT area, cm²** | 228.2 (179.9 – 290.2) | 182.1 (143.5 – 230.6) | -41.3 (-76.7 – -9.9) |
| **SSAT area, cm²** | 119.7 (91.5 – 162.9) | 96.5 (72.7 – 129.9) | -17.5 (-35.4 – -4.0) |
| **VAT proportion, %** | 28.2 ± 9.0 | 27.4 ± 9.1 | -1.3 ± 3.4 |
| **VAT/SAT** | 0.4 (0.3 – 0.5) | 0.4 (0.3 – 0.5) | -0.0 (-0.1 – 0.0) |
| **Anthropometric measurements** | | | |
| **Weight, Kg** | 92.6 ± 13.9 | 89.9 ± 14.3 | -2.6 ± 5.6 |
| **BMI, Kg/m²** | 30.9 ± 3.9 | 30.0 ± 4.1 | -0.9 ± 1.8 |
| **Waist circumference, cm** | 108.2 ± 9.7 | 103.5 ± 10.3 | -4.8 ± 5.9 |
| **Systolic, mmHg** | 125.5 (117.1 – 136.5) | 125.0 (116.0 – 135.0) | 0.0 (-7.5 – 7.0) |
| **Diastolic, mmHg** | 81.0 (74.0 – 87.0) | 79.0 (73.0 – 85.0) | -1.0 (-7.0 – 4.0) |
| **Physical Activity** | | | |
| **MET/week** | 24.7 (14.4 – 45.4) | 33.0 (18.8 – 62.8) | 7.3 (-5.9 – 26.2) |
| **Blood biomarkers** | | | |
| **Glucose, mg/dL** | 100.9 (94.4 – 108.7) | 101.0 (94.4 – 108.9) | -0.1 (-5.6 – 6.3) |
| **HbA1c, µU/mL** | 5.4 (5.2 – 5.7) | 5.4 (5.2 – 5.7) | 0.0 (-0.2 – 0.1) |
| **Fasting insulin, µU/mL** | 13.5 (10.2 – 19.0) | 11.6 (8.2 – 16.2) | -1.9 (-5.5 – 1.2) |
| **HOMA-IR** | 3.4 (2.5 – 4.9) | 3.0 (2.1 – 4.3) | -0.5 (-1.4 – 0.4) |
| **Triglycerides, mg/dL** | 138.1 (100.0 – 191.2) | 119.5 (92.7 – 171.7) | -7.1 (-37.6 – 14.6) |
| **HDLc, mg/dL** | 42.9 (35.9 – 51.0) | 45.6 (39.0 – 54.4) | 2.7 (-1.5 – 6.9) |
| **TG/HDL** | 3.2 (2.0 – 4.9) | 2.6 (1.7 – 4.2) | -0.3 (-1.2 – 0.3) |
| **GGT, U/L** | 30.6 (22.6 – 40.8) | 27.0 (19.8 – 37.2) | -2.4 (-8.4 – 1.8) |
| **ALT, U/L** | 27.6 (21.2 – 36.5) | 25.3 (19.7 – 33.5) | -1.8 (-7.6 – 2.9) |
| **AST, U/L** | 24.1 (20.6 – 30.0) | 23.5 (20.0 – 28.8) | -0.6 (-4.7 – 2.9) |
| **ALKP, U/L** | 70.0 (59.4 – 82.9) | 69.1 (58.2 – 81.6) | -1.8 (-7.6 – 4.1) |
| **Leptin, mg/mL** | 11.2 (6.7 – 17.9) | 8.3 (5.0 – 13.1) | -2.3 (-6.2 – 0.2) |
| **Chemerin, ng/mL** | 191.5 (175.3 – 215.8) | 188.3 (170.7 – 213.5) | 0.8 (-22.2 – 19.4) |
| **Ferritin, ng/mL** | 132.1 (76.9 – 210.0) | 126.0 (75.3 – 206.6) | -0.8 (-29.5 – 26.6) |
| **hsCRP, mg/L** | 2.5 (1.5 – 4.3) | 2.3 (1.3 – 4.1) | -0.1 (-1.2 – 0.7) |
| **Fetuin A, µg/mL** | 309.2 (259.2 – 367.4) | 271.6 (231.0 – 303.5) | -46.7 (-101.0 – 2.8) |

Values are presented as either median (p25 – p75) or mean ± standard deviation for continuous variables, depending on their distribution, and as number (%) for categorical variables.

VAT, visceral adipose tissue; SAT, subcutaneous adipose tissue; DSAT, deep subcutaneous adipose tissue; SSAT, superficial subcutaneous adipose tissue; HOMA-IR, homeostatic model assessment of insulin resistance; HDLc, high-density lipoprotein cholesterol; TG, Triglycerides; GGT, Gamma-glutamyl Transferase; ALKP, alkaline phosphatase; AST, aspartate transaminase; ALT, alanine transaminase; hsCRP, high sensitivity C reactive protein; MET, metabolic equivalent for task.

**Table S2.** **Baseline characteristics of the CENTRAL and DIRECT PLUS clinical trials participants across sex-specific deciles of visceral adipose tissue proportion. n=572 participants.**

| **Characteristics** | **Entire means pooled CENTRAL and DIRECT PLUS**  **N=572** | **VAT low**  **decile 1** | **VAT decile 2** | **VAT decile 3** | **VAT**  **decile 4** | **VAT**  **decile 5** | **VAT**  **decile 6** | **VAT**  **decile 7** | **VAT**  **decile 8** | **VAT**  **decile 9** | **VAT**  **top decile 10** | **Kendall's tau** | **P of trend^1^** | **FDR^2^** |
| --- | --- | --- | --- | --- | --- | --- | --- | --- | --- | --- | --- | --- | --- | --- |
| **Demographics** | | | | | | | | | | | | | | |
| **Sex** | | | | | | | | | | | | | | |
| ***Male*** | 506 (89.1) | 50 (86.2) | 49 (89.1) | 48 (88.9) | 49 (89.1) | 50 (89.3) | 48 (88.9) | 49 (89.1) | 48 (88.9) | 49 (89.1) | 50 (87.7) |  |  |  |
| ***Female*** | 62 (10.9) | 8 (13.8) | 6 (10.9) | 6 (11.1) | 6 (10.9) | 6 (10.7) | 6 (11.1) | 6 (10.9) | 6 (11.1) | 6 (10.9) | 7 (12.3) |  |  |  |
| **Age** | 49.5 ± 10.1 | 40.7 ± 7.0 | 43.2 ± 7.7 | 45.9 ± 8.6 | 48.5 ± 7.8 | 49.4 ± 8.8 | 48.3 ± 9.4 | 51.7 ± 8.8 | 51.4 ± 9.5 | 55.5 ± 9.2 | 58.9 ± 8.7 | 0.36 | **<0.001** | **<0.001** |
| **Anthropometric measurements** | | | | | | | | | | | | | | |
| **Weight, kg** | 92.6 ± 13.9 | 96.7 ± 12.7 | 94.7 ± 13.5 | 91.5 ± 12.2 | 95.2 ± 13.7 | 92.4 ± 12.9 | 90.9 ± 13.7 | 91.1 ± 11.8 | 91.1 ± 13.2 | 89.5 ± 11.2 | 88.3 ± 14.4 | -0.13 | **<0.001** | **<0.001** |
| **BMI, kg/m^2^** | 30.9 ± 3.9 | 31.9 ± 4.3 | 31.6 ± 4.1 | 30.9 ± 3.0 | 31.4 ± 3.5 | 30.7 ± 3.1 | 30.1 ± 4.0 | 30.5 ± 3.7 | 30.3 ± 3.4 | 30.5 ± 3.6 | 29.9 ± 3.9 | -0.11 | **<0.001** | **<0.001** |
| **Waist circumference, cm** | 108.2 ± 9.7 | 109.8 ± 9.6 | 107.6 ± 10.4 | 107.2 ± 7.8 | 109.4 ± 10.4 | 108.0 ± 7.7 | 106.4 ± 9.9 | 107.5 ± 7.5 | 107.1 ± 9.4 | 107.6 ± 7.5 | 107.6 ± 9.2 | -0.04 | 0.20 | 0.18 |
| **VAT area, cm^2^** | 134.8 (103.2 – 174.3) | 78.2 (65.4 – 93.2) | 103.2 (77.1 – 123.7) | 114.4 (97.6 – 135.3) | 134.3 (110.3 – 157.4) | 128.8 (106.8 – 163.8) | 124.6 (99.0 – 173.6) | 153.0 (120.6 – 178.0) | 162.0 (134.1 – 204.9) | 173.5 (158.2 – 204.0) | 210.6 (164.1 – 253.5) | 0.49 | **<0.001** | **<0.001** |
| **VAT/SAT** | 0.4 (0.3 – 0.5) | 0.2 (0.1 – 0.2) | 0.3 (0.2 – 0.3) | 0.3 (0.3 – 0.3) | 0.3 (0.3 – 0.4) | 0.4 (0.4 – 0.4) | 0.4 (0.4 – 0.4) | 0.5 (0.5 – 0.5) | 0.5 (0.5 – 0.5) | 0.6 (0.6 – 0.6) | 0.8 (0.7 – 0.9) | 0.83 | **<0.001** | **<0.001** |
| **Blood pressure** | | | | | | | | | | | | | | |
| **Systolic, mmHg** | 125.5 (117.1 – 136.5) | 121.3 (113.0 – 130.5) | 124.5 (118.0 – 131.0) | 122.5 (116.0 – 130.0) | 123.8 (114.3 – 130.9) | 127.3 (120.4 – 138.5) | 124.3 (116.1 – 132.9) | 126.0 (115.5 – 136.8) | 124.5 (113.6 – 132.8) | 137.0 (123.5 – 145.5) | 135.5 (124.0 – 147.0) | 0.16 | **<0.001** | **<0.001** |
| **Diastolic, mmHg** | 81.0 (74.0 – 87.0) | 76.5 (71.3 – 83.9) | 80.0 (74.0 – 84.8) | 80.0 (73.0 – 85.0) | 79.0 (73.6 – 84.9) | 82.8 (77.0 – 87.1) | 79.0 (71.1 – 85.9) | 82.0 (73.5 – 86.8) | 79.3 (74.5 – 87.0) | 85.5 (76.5 – 93.0) | 82.5 (72.0 – 92.5) | 0.10 | **<0.001** | **0.002** |
| **Metabolic complications** | | | | | | | | | | | | | | |
| **Diabetes** | | | | | | | | | | | | | | |
| ***Non-diabetes*** | 506 (89.1) | 52 (91.2) | 51 (94.4) | 45 (86.5) | 51 (92.7) | 50 (89.3) | 49 (90.7) | 52 (94.5) | 50 (92.6) | 45 (81.8) | 42 (73.7) |  |  |  |
| ***Diabetes*** | 62 (10.9) | 5 (8.8) | 3 (5.6) | 7 (13.5) | 4 (7.3) | 6 (10.7) | 5 (9.3) | 3 (5.5) | 4 (7.4) | 10 (18.2) | 15 (26.3) |  |  |  |
| **Metabolic syndrome** | | | | | | | | | | | | | | |
| ***No metabolic syndrome*** | 209 (37.4) | 33 (57.9) | 27 (50.9) | 22 (43.1) | 27 (50.9) | 17 (30.4) | 21 (39.6) | 18 (32.7) | 20 (38.5) | 11 (20.0) | 8 (14.5) |  |  |  |
| ***Metabolic syndrome*** | 350 (62.6) | 24 (42.1) | 26 (49.1) | 29 (56.9) | 26 (49.1) | 39 (69.6) | 32 (60.4) | 37 (67.3) | 32 (61.5) | 44 (80.0) | 47 (85.5) |  |  |  |
| **Blood biomarkers** | | | | | | | | | | | | | | |
| **Glucose, mg/dL** | 100.9 (94.4 – 108.7) | 99.1 (91.9 – 103.8) | 96.9 (92.6 – 103.8) | 100.6 (94.4 – 105.7) | 100.9 (91.6 – 107.5) | 102.5 (95.9 – 107.4) | 97.2 (92.6 – 107.2) | 98.4 (94.5 – 106.8) | 99.3 (96.0 – 106.8) | 104.7 (98.1 – 116.2) | 109.9 (100.9 – 122.5) | 0.16 | **<0.001** | **<0.001** |
| **HbA1c, %** | 5.4 (5.2 – 5.7) | 5.2 (5.0 – 5.5) | 5.4 (5.2 – 5.6) | 5.4 (5.1 – 5.6) | 5.4 (5.2 – 5.7) | 5.4 (5.2 – 5.8) | 5.4 (5.1 – 5.7) | 5.4 (5.2 – 5.7) | 5.4 (5.3 – 5.6) | 5.7 (5.4 – 6.0) | 5.6 (5.3 – 5.9) | 0.16 | **<0.001** | **<0.001** |
| **Fasting insulin, µU/mL** | 13.5 (10.2 – 19.0) | 11.4 (8.1 – 14.8) | 12.9 (9.8 – 16.0) | 13.4 (10.4 – 17.2) | 14.5 (9.5 – 23.8) | 14.2 (11.0 – 20.1) | 13.5 (11.0 – 17.8) | 13.3 (9.4 – 18.4) | 14.5 (10.1 – 19.3) | 13.8 (11.3 – 19.6) | 16.2 (12.7 – 20.9) | 0.10 | **0.001** | **0.002** |
| **HOMA-IR^2^** | 3.4 (2.5 – 4.9) | 2.7 (2.0 – 3.7) | 3.1 (2.4 – 4.0) | 3.4 (2.4 – 4.4) | 3.7 (2.5 – 6.3) | 3.4 (2.6 – 5.0) | 3.2 (2.6 – 4.7) | 3.3 (2.2 – 4.8) | 3.8 (2.4 – 5.2) | 3.8 (2.8 – 5.5) | 4.3 (3.2 – 5.8) | 0.12 | **<0.001** | **<0.001** |
| **Triglycerides, mg/dL** | 138.1 (100.0 – 191.2) | 106.2 (87.4 – 157.3) | 129.2 (93.4 – 166.4) | 142.9 (110.8 – 222.8) | 144.7 (101.1 – 199.8) | 130.5 (97.1 – 179.2) | 134.5 (88.5 – 190.3) | 138.9 (104.0 – 192.9) | 137.2 (110.4 – 200.0) | 148.7 (121.2 – 213.7) | 168.2 (127.0 – 224.3) | 0.12 | **<0.001** | **<0.001** |
| **HDLc, mg/dL** | 44.6 ± 11.7 | 47.1 ± 11.0 | 45.7 ± 13.5 | 44.0 ± 12.4 | 45.0 ± 12.0 | 45.9 ± 11.9 | 44.9 ± 12.3 | 42.0 ± 12.4 | 43.5 ± 9.8 | 43.0 ± 9.6 | 44.1 ± 13.0 | -0.07 | **0.02** | **0.03** |
| **Triglycerides/HDLc** | 3.2 (2.0 – 4.9) | 2.3 (1.6 – 3.5) | 2.9 (2.0 – 4.6) | 3.3 (2.1 – 5.3) | 3.4 (1.9 – 5.3) | 3.1 (2.0 – 4.5) | 3.4 (1.7 – 5.2) | 3.5 (2.4 – 5.2) | 3.2 (2.3 – 5.4) | 4.1 (2.4 – 5.5) | 4.0 (3.0 – 5.7) | 0.12 | **<0.001** | **<0.001** |
| **GGT, U/L** | 30.6 (22.6 – 40.8) | 24.6 (19.8 – 33.9) | 27.6 (21.0 – 37.8) | 27.0 (18.3 – 42.0) | 30.9 (22.3 – 38.2) | 32.4 (26.4 – 43.3) | 28.8 (20.2 – 49.6) | 35.4 (22.8 – 46.2) | 31.2 (23.4 – 36.6) | 33.9 (26.1 – 45.3) | 34.2 (28.2 – 45.6) | 0.12 | **<0.001** | **<0.001** |
| **ALT, U/L** | 27.6 (21.2 – 36.5) | 27.1 (20.1 – 38.1) | 24.7 (17.6 – 34.1) | 24.7 (20.0 – 34.0) | 27.6 (22.2 – 36.6) | 30.0 (23.5 – 35.9) | 25.9 (20.0 – 34.1) | 27.1 (21.5 – 42.1) | 26.5 (19.4 – 35.9) | 26.8 (23.5 – 32.6) | 29.4 (25.0 – 38.2) | 0.05 | 0.10 | 0.12 |
| **AST, U/L** | 24.1 (20.6 – 30.0) | 25.6 (20.7 – 31.0) | 23.5 (20.0 – 27.6) | 22.4 (19.4 – 27.1) | 25.0 (20.6 – 31.2) | 25.3 (21.5 – 30.3) | 24.1 (20.0 – 28.8) | 24.1 (20.9 – 31.2) | 24.4 (20.0 – 29.4) | 24.4 (21.2 – 29.3) | 24.7 (20.1 – 30.3) | 0.01 | 0.70 | 0.70 |
| **ALKP, U/L** | 70.0 (59.4 – 82.9) | 67.4 (60.0 – 79.1) | 66.5 (58.5 – 79.4) | 69.1 (58.5 – 82.8) | 65.3 (54.3 – 82.5) | 72.9 (61.8 – 83.2) | 65.9 (57.6 – 80.0) | 78.8 (60.9 – 91.5) | 75.3 (62.2 – 87.8) | 70.0 (60.0 – 84.1) | 70.0 (63.1 – 82.2) | 0.07 | **0.02** | **0.02** |
| **Leptin, ng/mL** | 11.2 (6.7 – 17.9) | 13.5 (9.1 – 22.0) | 12.1 (7.4 – 18.6) | 10.7 (8.5 – 16.9) | 13.8 (10.3 – 19.2) | 11.2 (5.9 – 18.5) | 8.9 (6.2 – 13.8) | 9.1 (6.2 – 15.5) | 13.6 (6.8 – 18.3) | 7.6 (6.3 – 11.9) | 8.8 (6.3 – 14.8) | -0.12 | **<0.001** | **<0.001** |
| **Chemerin, ng/mL** | 191.5 (175.3 – 215.8) | 183.6 (171.2 – 204.1) | 187.0 (164.3 – 207.6) | 187.0 (177.6 – 215.9) | 199.9 (183.8 – 216.6) | 190.2 (174.3 – 217.1) | 182.3 (168.9 – 196.4) | 197.1 (172.1 – 217.3) | 192.7 (180.5 – 211.8) | 192.5 (174.9 – 227.4) | 200.6 (179.1 – 228.3) | 0.08 | **0.01** | **0.02** |
| **Ferritin, ng/mL** | 132.1 (76.9 – 210.0) | 101.7 (67.2 – 170.3) | 128.0 (67.4 – 171.7) | 117.6 (75.1 – 197.5) | 133.0 (76.2 – 224.5) | 174.0 (102.6 – 253.0) | 123.0 (74.9 – 168.3) | 170.9 (87.7 – 245.0) | 144.0 (77.2 – 231.9) | 116.0 (71.1 – 178.7) | 142.0 (103.8 – 187.8) | 0.05 | 0.10 | 0.12 |
| **Fetuin A, µg/mL** | 309.2 (257.6 – 367.4) | 308.1 (264.5 – 368.2) | 295.4 (256.9 – 368.8) | 306.0 (252.1 – 361.0) | 322.3 (269.6 – 361.5) | 331.2 (266.1 – 385.4) | 311.5 (271.7 – 358.3) | 293.5 (247.4 – 341.1) | 287.9 (246.8 – 332.8) | 308.8 (250.4 – 383.4) | 330.9 (282.6 – 400.4) | 0.02 | 0.60 | 0.64 |
| **hsCRP, mg/L** | 2.5 (1.5 – 4.3) | 2.8 (1.5 – 4.2) | 2.2 (1.3 – 3.9) | 2.7 (1.3 – 4.2) | 2.2 (1.5 – 4.0) | 3.0 (1.9 – 5.0) | 2.3 (1.4 – 3.7) | 2.6 (1.6 – 4.1) | 3.1 (1.5 – 5.6) | 2.8 (1.6 – 4.7) | 2.1 (1.6 – 4.0) | 0.02 | 0.50 | 0.57 |

Values are presented as either median (p25 – p75) or mean ± standard deviation for continuous variables, depending on their distribution, and as number (%) for categorical variables.

VAT, visceral adipose tissue; SAT, subcutaneous adipose tissue; HOMA-IR, homeostatic model assessment of insulin resistance; hsCRP, high sensitivity C reactive protein; HDLc, high-density lipoprotein cholesterol; GGT, Gamma-glutamyl Transferase; ALKP, alkaline phosphatase; AST, aspartate transaminase; ALT, alanine transaminase.

^1^ P of trend was analyzed using Kendall tau correlation test.

^2^ False discovery rate correction for multiple testing

**
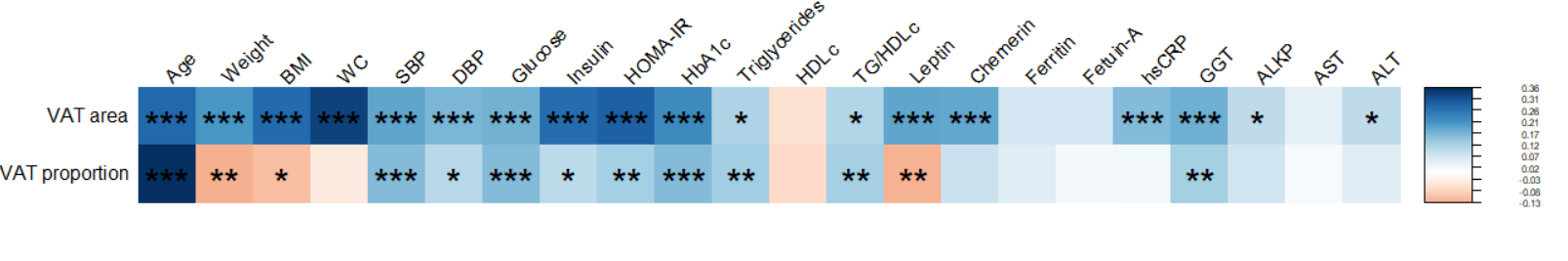
****Figure S1.** **Baseline visceral abdominal adipose tissue area and proportion** **across various** **characteristics; Heatmap of Kendall's tau correlations.** Baseline characteristics across visceral abdominal adipose tissue sex-specific ranks. Correlations are color-coded according to the direction of the effect (blue =positive correlation, red=negative correlation). Benjamini-Hochberg correction was used for multiple comparisons (FDR 5%). Asterisk number (***, **, *) corresponds to p-values of 0.001, 0.01, and 0.05. VAT, visceral adipose tissue; WC, waist circumference; SBP, systolic blood pressure; DBP, diastolic blood pressure; HOMA-IR, homeostatic model assessment of insulin resistance; hsCRP, high sensitivity C reactive protein; HDLc, high-density lipoprotein cholesterol; TG, Triglycerides; GGT, Gamma-glutamyl Transferase; ALKP, alkaline phosphatase; AST, aspartate transaminase; ALT, alanine transaminase.

**Table S3.** **Visceral adiposity phenotypes of similar and opposite sex-specific visceral adipose tissue area and proportion medians. N=553.**

|  | **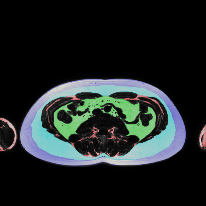** | 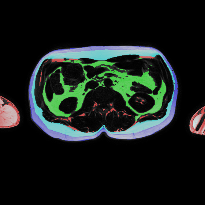 | 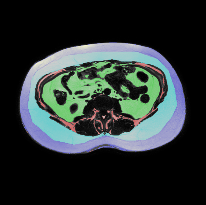 | 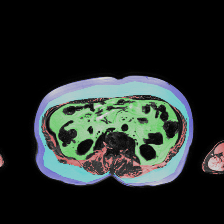 |  |  |
| --- | --- | --- | --- | --- | --- | --- |
| **Characteristic** | **Low-median VAT area and low-median VAT proportion**  **N = 203 (37%)** | **Low-median VAT area and top-median VAT proportion**  **N = 78 (14%)** | **Top-median VAT area and low-median VAT proportion**  **N = 72 (13%)** | **Top-median VAT area and top-median VAT proportion**  **N = 200 (36%)** | **^1^P of trend, adjusted to weight and DSAT** | **^2^P between groups, adjusted to weight and DSAT** |
| VAT area, cm² | 98.7 ± 26.4 | 112.1 ± 21.1 | 163.0 ± 32.3 | 197.0 ± 53.8 | <0.001 | <0.001 |
| VAT proportion, % | 20.4 ± 5.1 | 31.0 ± 5.1 | 24.4 ± 3.9 | 36.3 ± 6.8 | <0.001 | <0.001 |
| VAT/SAT | 0.3 ± 0.1 | 0.5 ± 0.1 | 0.3 ± 0.1 | 0.6 ± 0.2 | <0.001 | <0.001 |
| Sex (% male) | 180 (88.7) | 69 (88.5) | 64 (88.9) | 177 (88.5) |  |  |
| Weight, Kg | 91.2 ± 11.6 | 82.8 ± 9.8 | 102.7 ± 12.6 | 93.0 ± 13.0 |  |  |
| BMI, Kg/m² | 30.4 ± 3.0 | 27.7 ± 2.4 | 33.9 ± 3.9 | 31.3 ± 3.7 |  |  |
| WC, cm | 106.1 ± 8.1 | 100.8 ± 6.4 | 115.6 ± 8.3 | 109.6 ± 8.3 | <0.001 | <0.001 |
| SSAT area, cm² | 147.3 ± 58.9 | 92.0 ± 28.9 | 181.7 ± 71.2 | 124.5 ± 61.1 | <0.001 | <0.001 |
| DSAT area, cm² | 248.6 ± 66.0 | 162.4 ± 39.3 | 335.6 ± 79.7 | 231.4 ± 72.0 |  |  |
| Age | 44.6 ± 8.5 | 50.0 ± 10.1 | 47.6 ± 8.4 | 54.5 ± 9.3 | <0.001 | <0.001 |
| Systolic, mmHg | 123.8 ± 14.0 | 124.6 ± 14.5 | 127.8 ± 12.2 | 131.0 ± 17.0 | <0.001 | <0.001 |
| Diastolic, mmHg | 79.0 ± 9.6 | 78.1 ± 11.8 | 82.4 ± 8.0 | 81.8 ± 11.0 | <0.001 | 0.003 |
| Glucose, mg/dL | 101.5 ± 17.6 | 102.3 ± 13.8 | 104.3 ± 15.2 | 110.9 ± 28.4 | <0.001 | <0.001 |
| HbA1c, µU/mL | 5.4 ± 0.5 | 5.4 ± 0.6 | 5.6 ± 0.4 | 5.7 ± 0.7 | <0.001 | <0.001 |
| Insulin, µU/mL | 13.8 ± 7.9 | 11.7 ± 5.9 | 20.3 ± 11.7 | 17.5 ± 8.8 | <0.001 | <0.001 |
| HOMA-IR | 3.5 ± 2.5 | 3.0 ± 1.6 | 5.2 ± 3.2 | 4.8 ± 2.9 | <0.001 | <0.001 |
| Triglycerides, mg/dL | 144.7 ± 73.3 | 153.5 ± 86.6 | 170.1 ± 105.2 | 171.1 ± 89.2 | <0.001 | 0.018 |
| HDLc, mg/dL | 46.2 ± 12.0 | 44.6 ± 13.0 | 43.6 ± 12.2 | 43.1 ± 11.0 | 0.007 | 0.078 |
| TG/HDLc | 3.6 ± 2.7 | 4.0 ± 3.1 | 4.6 ± 4.2 | 4.5 ± 3.5 | <0.001 | 0.032 |
| Total cholesterol, mg/dL | 194.6 ± 34.4 | 190.1 ± 34.6 | 201.2 ± 40.2 | 198.9 ± 36.9 | 0.117 | 0.194 |
| LDLc, mg/dL | 123.9 ± 29.1 | 118.7 ± 32.2 | 125.2 ± 29.4 | 125.7 ± 33.1 | 0.363 | 0.429 |
| GGT, U/L | 32.0 ± 20.6 | 38.7 ± 31.4 | 36.9 ± 14.4 | 39.5 ± 22.6 | <0.001 | 0.011 |
| ALT, U/L | 30.0 ± 18.7 | 27.7 ± 12.9 | 33.1 ± 17.8 | 31.9 ± 14.6 | 0.028 | 0.159 |
| AST, U/L | 26.3 ± 11.6 | 24.9 ± 7.6 | 29.1 ± 11.7 | 26.4 ± 8.5 | 0.148 | 0.104 |
| ALKP, mg/dL | 70.0 ± 17.9 | 73.1 ± 19.7 | 74.3 ± 19.4 | 74.5 ± 21.8 | 0.006 | 0.141 |
| Leptin, ng/mL | 13.5 ± 9.9 | 8.0 ± 6.5 | 21.3 ± 14.8 | 14.6 ± 12.9 | 0.031 | <0.001 |
| Chemerin, ng/mL | 193.0 ± 32.7 | 186.5 ± 31.9 | 210.3 ± 43.9 | 203.6 ± 35.0 | <0.001 | <0.001 |
| Ferritin, ng/mL | 151.6 ± 110.3 | 154.1 ± 131.9 | 155.5 ± 102.8 | 157.7 ± 97.0 | 0.210 | 0.954 |
| hsCRP, mg/L | 3.4 ± 4.7 | 3.5 ± 5.9 | 4.3 ± 2.7 | 4.4 ± 5.7 | <0.001 | 0.228 |
| Fetuin A, µg/mL | 323.8 ± 93.7 | 308.2 ± 88.5 | 324.9 ± 78.0 | 328.0 ± 89.2 | 0.449 | 0.449 |

^1^Kendall tau + FDR correction for multiple testing

^2^ANCOVA + FDR correction for multiple testing

Values are presented as mean ± standard deviation for continuous variables and number (%) for categorical variables.

VAT, visceral adipose tissue; SAT, subcutaneous adipose tissue; WC, waist circumference; DSAT, deep subcutaneous adipose tissue; SSAT, superficial subcutaneous adipose tissue; HOMA-IR, homeostatic model assessment of insulin resistance; TG, Triglycerides; hsCRP, high sensitivity C reactive protein; HDLc, high-density lipoprotein cholesterol; GGT, Gamma-glutamyl Transferase; ALKP, alkaline phosphatase; AST, aspartate transaminase; ALT, alanine transaminase.

**Table S4.** **Post hoc analysis of visceral adiposity phenotypes of similar and opposite sex-specific visceral adipose tissue area and proportion medians. N=553.**

| **Characteristic** | ^1^**Low-median VAT area and low-median VAT proportion vs. Low-median VAT area and top-median VAT proportion** | ^1^**Low-median VAT area and low-median VAT proportion vs. Top-median VAT area and low-median VAT proportion** | ^1^**Low-median VAT area and top-median VAT proportion vs. Top-median VAT area and low-median VAT proportion** | ^1^**Low-median VAT area and low-median VAT proportion vs. Top-median VAT area and top-median VAT proportion** | ^1^**Low-median VAT area and top-median VAT proportion vs. Top-median VAT area and top-median VAT proportion** | ^1^**Top-median VAT area and low-median VAT proportion vs. Top-median VAT area and top-median VAT proportion** |
| --- | --- | --- | --- | --- | --- | --- |
| **VAT proportion, %** | <0.001 | <0.001 | <0.001 | <0.001 | <0.001 | <0.001 |
| **VAT area, cm²** | <0.001 | <0.001 | <0.001 | <0.001 | <0.001 | <0.001 |
| **VAT/SAT** | <0.001 | <0.001 | <0.001 | <0.001 | <0.001 | <0.001 |
| **Weight, Kg** | <0.001 | <0.001 | <0.001 | 0.13 | <0.001 | <0.001 |
| **BMI, Kg/m²** | <0.001 | <0.001 | <0.001 | 0.008 | <0.001 | <0.001 |
| **Waist circumference, cm** | <0.001 | <0.001 | <0.001 | <0.001 | <0.001 | <0.001 |
| **SSAT area, cm²** | <0.001 | <0.001 | <0.001 | <0.001 | <0.001 | <0.001 |
| **DSAT area, cm²** | <0.001 | <0.001 | <0.001 | 0.002 | <0.001 | <0.001 |
| **Age** | <0.001 | 0.033 | 0.10 | <0.001 | <0.001 | <0.001 |
| **Systolic, mmHg** | 0.6 | 0.087 | 0.3 | <0.001 | 0.014 | 0.3 |
| **Diastolic, mmHg** | >0.9 | 0.021 | 0.059 | 0.010 | 0.059 | >0.9 |
| **Glucose, mg/dL** | 0.6 | 0.10 | 0.6 | <0.001 | 0.055 | 0.6 |
| **HbA1c, µU/mL** | >0.9 | <0.001 | 0.004 | <0.001 | <0.001 | >0.9 |
| **Fasting insulin, µU/mL** | 0.086 | <0.001 | <0.001 | <0.001 | <0.001 | 0.2 |
| **HOMA-IR** | 0.2 | <0.001 | <0.001 | <0.001 | <0.001 | 0.3 |
| **Triglycerides, mg/dL** | >0.9 | 0.4 | >0.9 | 0.002 | 0.3 | >0.9 |
| **HDLc, mg/dL** | 0.5 | 0.3 | >0.9 | 0.025 | >0.9 | >0.9 |
| **Triglycerides/HDLc** | 0.7 | 0.2 | >0.9 | <0.001 | 0.6 | >0.9 |
| **GGT, U/L** | >0.9 | 0.004 | 0.2 | <0.001 | 0.11 | >0.9 |
| **ALT, U/L** | >0.9 | 0.2 | 0.10 | 0.12 | 0.093 | >0.9 |
| **AST, U/L** | 0.7 | 0.2 | 0.2 | 0.6 | 0.6 | 0.6 |
| **ALKP, mg/dL** | >0.9 | 0.5 | >0.9 | 0.063 | >0.9 | >0.9 |
| **Leptin, ng/mL** | <0.001 | <0.001 | <0.001 | 0.7 | <0.001 | <0.001 |
| **Chemerin, ng/mL** | 0.2 | 0.004 | <0.001 | 0.003 | <0.001 | 0.3 |
| **hsCRP, mg/L** | 0.2 | <0.001 | <0.001 | 0.014 | 0.002 | 0.10 |

^1^Post hoc analyses were performed via either un-paired t-test or Mann-Whitney U test, depending on variables distribution, with a Bonferroni correction for multiple comparisons

VAT, visceral adipose tissue; SAT, subcutaneous adipose tissue; WC, waist circumference; DSAT, deep subcutaneous adipose tissue; SSAT, superficial subcutaneous adipose tissue; HOMA-IR, homeostatic model assessment of insulin resistance; TG, Triglycerides; hsCRP, high sensitivity C reactive protein; HDLc, high-density lipoprotein cholesterol; GGT, Gamma-glutamyl Transferase; ALKP, alkaline phosphatase; AST, aspartate transaminase; ALT, alanine transaminase.

**Table S5. LASSO developed models for baseline VAT area.**

|  | **Selected variables** | **Train RMSE*** | **Train R^2^*** | **Test RMSE**** | **Test R^2^**** | **Validation RMSE***** | **Validation R^2^***** |
| --- | --- | --- | --- | --- | --- | --- | --- |
| **Anthropometrics and demographics** | WC, MAP, Age, Sex | 0.29 | 0.41 | 0.27 | 0.5 | 0.41 | 0.45 |
| **Blood biomarkers** | TG/HDLc, HbA1c, HOMA-IR, Glucose, GGT, ALKP, AST, Leptin, Chemerin, Ferritin, hsCRP | 0.33 | 0.17 | 0.34 | 0.22 | 0.38 | 0.24 |
| **Anthropometrics, demographics, and blood biomarkers** | WC, MAP, Age, TG/HDLc, HbA1c, HOMA-IR, Glucose, GGT, ALKP, Chemerin | 0.27 | 0.44 | 0.26 | 0.53 | 0.4 | 0.5 |

* Performance of 100 (10-fold*10 repeats) cross-validation models.

** Performance of final model on testing data.

*** Performance of final model on validation data.

VAT, visceral adipose tissue; WC, waist circumference; MAP, mean arterial pressure; HOMA-IR, homeostatic model assessment of insulin resistance; hsCRP, high sensitivity C reactive protein; HDLc, high-density lipoprotein cholesterol; TG, Triglycerides; GGT, Gamma-glutamyl Transferase; ALKP, alkaline phosphatase; AST, aspartate transaminase.

**Table S6. LASSO developed models for baseline VAT proportion.**

|  | **Selected variables** | **Train RMSE*** | **Train R^2^*** | **Test RMSE**** | **Test R^2^**** | **Validation RMSE***** | **Validation R^2^** |
| --- | --- | --- | --- | --- | --- | --- | --- |
| **Anthropometrics and demographics** | Weight, MAP, Age, Sex | 7.38 | 0.31 | 7.03 | 0.4 | 7.44 | 0.33 |
| **Blood biomarkers** | LDLc, Total cholesterol, Triglycerides, HbA1c, HOMA-IR, Glucose, ALKP, AST, Leptin, Chemerin, Ferritin, Fetuin A, hsCRP | 7.72 | 0.21 | 7.63 | 0.28 | 7.9 | 0.18 |
| **Anthropometrics, demographics, and blood biomarkers** | MAP, Age, Sex, TG/HDLc, HOMA-IR, Glucose, GGT, ALKP, ALT, Leptin, Chemerin, Ferritin, Fetuin A | 6.87 | 0.37 | 6.55 | 0.51 | 6.7 | 0.39 |

* Performance of 100 (10-fold*10 repeats) cross-validation models.

** Performance of final model on testing data.

*** Performance of final model on validation data.

VAT, visceral adipose tissue; WC, waist circumference; MAP, mean arterial pressure; HOMA-IR, homeostatic model assessment of insulin resistance; hsCRP, high sensitivity C reactive protein; HDLc, high-density lipoprotein cholesterol; TG, Triglycerides; GGT, Gamma-glutamyl Transferase; ALKP, alkaline phosphatase; AST, aspartate transaminase; ALT, alanine transaminase.

**Table S7. LASSO developed models for VAT area relative change.**

|  | **Selected variables** | **Train RMSE*** | **Train R^2^*** | **Test RMSE**** | **Test R^2^**** | **Validation RMSE***** | **Validation R^2^** |
| --- | --- | --- | --- | --- | --- | --- | --- |
| **Anthropometrics and demographics** | Weight, WC, MAP | 15.54 | 0.52 | 15.33 | 0.59 | 52.94 | 0.44 |
| **Blood biomarkers** | Triglycerides, HDLc, HbA1c, ALT, hsCRP, Leptin, Chemerin, Ferritin, Fetuin A, LDLc | 18.38 | 0.33 | 16.5 | 0.5 | 56.86 | 0.12 |
| **Anthropometrics, demographics, and blood biomarkers** | Weight, WC, Leptin | 15.59 | 0.53 | 15.2 | 0.55 | 52.1 | 0.52 |

* Performance of 100 (10-fold*10 repeats) cross-validation models.

** Performance of final model on testing data.

*** Performance of final model on validation data.

VAT, visceral adipose tissue; WC, waist circumference; MAP, mean arterial pressure; HDLc, high-density lipoprotein cholesterol; ALT, alanine transaminase.

**Table S8. LASSO developed models for VAT proportion relative change.**

|  | **Selected variables** | **Train RMSE*** | **Train R^2^*** | **Test RMSE**** | **Test R^2^**** | **Validation RMSE***** | **Validation R^2^** |
| --- | --- | --- | --- | --- | --- | --- | --- |
| **Anthropometrics and demographics** | Weight, WC, MAP, Age, Sex | 11.32 | 0.15 | 11.8 | 0.24 | 18.72 | 0.16 |
| **Blood biomarkers** | Triglycerides, HDLc, HbA1c, AST, Leptin, Chemerin, Ferritin | 11.79 | 0.14 | 11.97 | 0.01 | 18.74 | 0.05 |
| **Anthropometrics, demographics, and blood biomarkers** | Weight, WC, MAP, Sex, Triglycerides, HDLc, AST, Chemerin, Ferritin | 11.81 | 0.14 | 11.91 | 0.06 | 18.9 | 0.16 |

* Performance of 100 (10-fold*10 repeats) cross-validation models.

** Performance of final model on testing data.

*** Performance of final model on validation data.

VAT, visceral adipose tissue; WC, waist circumference; MAP, mean arterial pressure; HDLc, high-density lipoprotein cholesterol; AST, aspartate transaminase.

**Table S9. Participants’ characteristics in the training and testing data sets for baseline VAT area model development.**


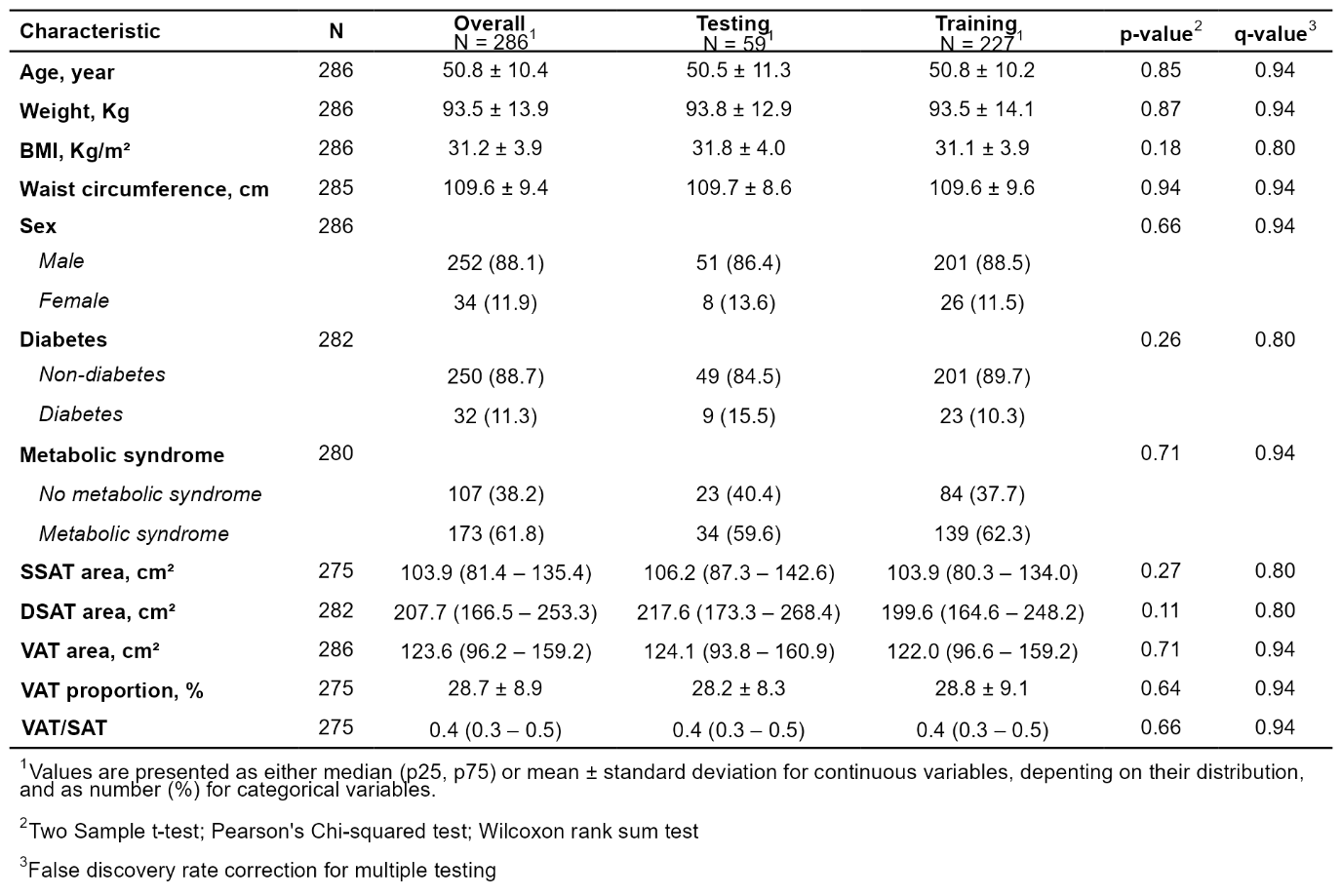


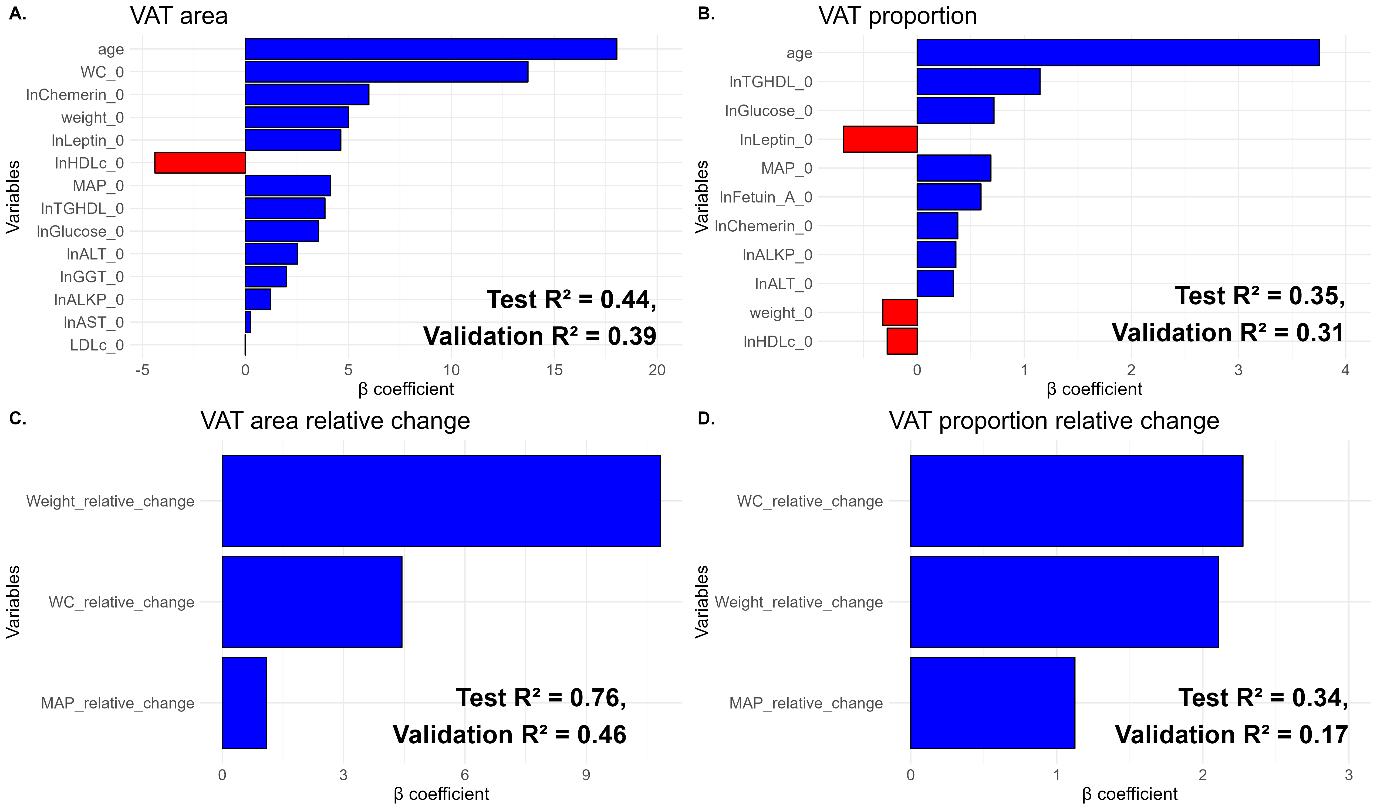


1. ***VAT area*** = 135.35 + 18.04*Age (years) + 13.72*WC (cm) + 6.00*ln(Chemerin) (ng/mL) + 5.00*Weight (Kg) + 4.63*ln(Leptin) - 4.40*ln(HDLc) (mg/dL) + 4.12*MAP (mmHg) + 3.86*ln(TG/HDLc) + 3.55*ln(Glucose) (mg/dL) + 2.53*ln(ALT) (U/L) + 1.98*ln(GGT) (U/L) + 1.22*ln(ALKP) (U/L) + 0.235*ln(AST) (U/L) - 0.02*LDLc (mg/dL)
2. ***VAT proportion =*** 29.97 + 3.75*Age (years) + 1.15*ln(TG/HDLc) + 0.72*ln(Glucose) (mg/dL) - 0.69*ln(Leptin) (ng/mL) + 0.69*MAP (mmHg) + 0.56*ln(Fetuin_A) (µg/mL) + 0.38*ln(Chemerin) (ng/mL) + 0.361*ln(ALKP) (U/L) + 0.34*ln(ALT) (U/L) - 0.32*Weight (Kg) - 0.28*HDLc (mg/dL)
3. ***VAT area relative change*** = -10.52 + 10.83*Weight_relative_change (%) + 4.44*WC_relative_change (%) + 1.09*MAP_relative_change (%)
4. ***VAT proportion relative change*** = -3.80 + 2.27*WC_relative_change + 2.11*Weight_relative_change (%) + 1.12*MAP_relative_change (%)

**Figure S2.** **LASSO linear regression models of baseline and change VAT area and proportion (men only).** The x-axis displays the variables selected by the LASSO model and the y-axis represents the estimated β-unstandardized coefficients. The magnitude and direction by which each variable affects baseline VAT area (A) and proportion (B), and changes of VAT area (C) and proportion (D) are represented by the color (blue for positive and red for negative associations) and length of the bars. VAT, visceral adipose tissue. Baseline VAT area (A) model was trained on a set of n=200 participants, tested on n=43 participants and validated on 116 participants. Baseline VAT proportion (B) model was trained on a set of n=192 participants, tested on n=49 participants and validated on n=120 participants. VAT area change (C) model was trained on a set of n=160 participants, tested on n=41 participants and validated on n=194 participants. VAT proportion change (D) model was trained on a set of n=153 participants, tested on n=33 participants and validated on n=100 participants. Abbreviations: LASSO, Least Absolute Shrinkage and Selection Operator; VAT, Visceral Adipose Tissue; WC, Waist Circumference; MAP, Mean Arterial Pressure; TG, Triglycerides; HDLc, High-Density Lipoprotein cholesterol; HOMA-IR, Homeostatic Model Assessment of Insulin Resistance; GGT, Gamma-Glutamyl Transferase; AST, Aspartate Transaminase; ALKP, alkaline phosphatase; ALT, alanine transaminase.


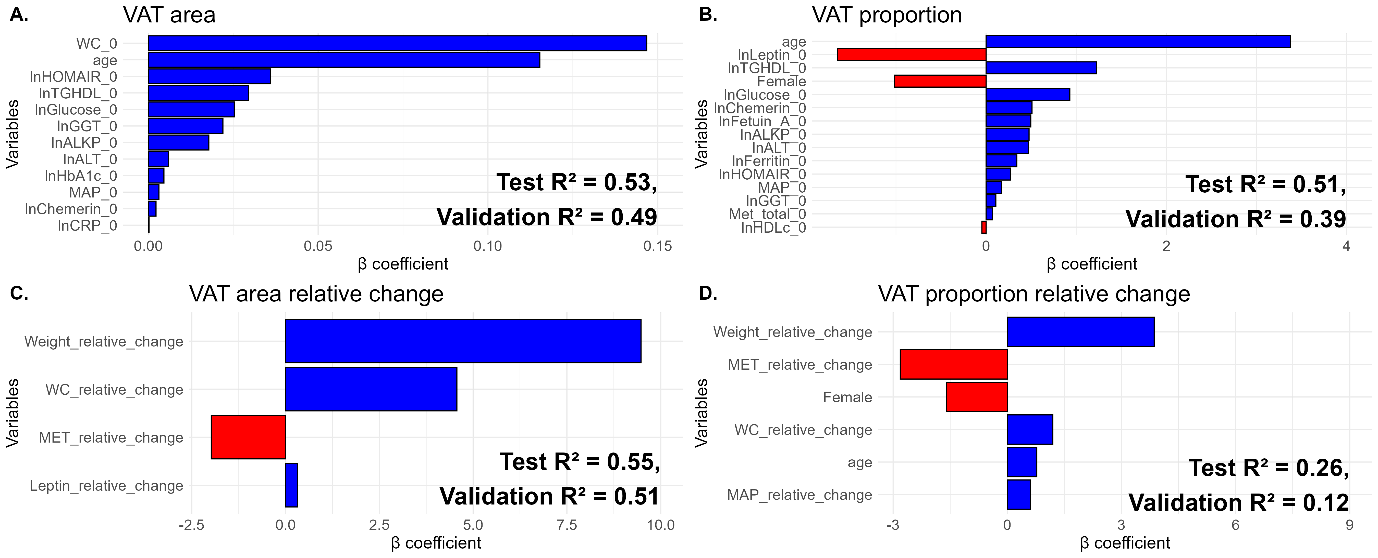


1. ***ln VAT area*** = 4.817 + 0.147*WC (cm) + 0.115*Age (years) + 0.036*ln(HOMA-IR) + 0.03*ln(TG/HDLc) + 0.025*ln(Glucose) (mg/dL) + 0.022*ln(GGT) (U/L) + 0.018*ln(ALKP) (U/L) + 0.006*ln(ALT) (U/L) + 0.005*ln(HbA1c) (%) + 0.003*MAP (mmHg) + 0.002*ln(Chemerin) (ng/mL)
2. ***VAT proportion =*** 28.81 + 3.377*Age (years) – 1.651*ln(Leptin) (ng/mL) + 1.225*ln(TG/HDLc) – 1.018*Sex (Male=0, Female=1) + 0.928*ln(Glucose) (mg/dL) + 0.509*ln(Chemerin) (ng/mL) + 0.495*ln(Fetuin_A) + 0.476*ln(ALKP) (U/L) + 0.471*ln(ALT) (U/L) + 0.34*ln(Ferritin) (ng/mL) + 0.269*ln(HOMA-IR) + 0.172*MAP (mmHg) + 0.108*ln(GGT) (U/L) + 0.071 (MET/week) - 0.047*HDLc (mg/dL)
3. ***VAT area relative change*** = -10.148 + 9.476*Weight_relative_change (%) + 4.567*WC_relative_change (%) – 1.98*MET/week_relative_change (%) + 0.319*Leptin_relative_change (%)
4. ***VAT proportion relative change*** = -3.949 + 3.862*Weight_relative_change (%) – 2.812*MET_relative_change (%) – 1.599*Sex (Male=0, Female=1) + 1.196*WC_relative_change (%) + 0.775*Age (years) + 0.605*MAP_relative_change (%)

**Figure S3. LASSO linear regression models of baseline and change VAT area and proportion while accounting for physical activity.** The x-axis displays the variables selected by the LASSO model and the y-axis represents the estimated β-unstandardized coefficients. The magnitude and direction by which each variable affects baseline VAT area (A) and proportion (B), and changes of VAT area (C) and proportion (D) are represented by the color (blue for positive and red for negative associations) and length of the bars. VAT, visceral adipose tissue. Baseline VAT area (A) model was trained on a set of n=227 participants, tested on n=55 participants and validated on 143 participants. Baseline VAT proportion (B) model was trained on a set of n=218 participants, tested on n=52 participants and validated on n=139 participants. VAT area change (C) model was trained on a set of n=180 participants, tested on n=44 participants and validated on n=195 participants. VAT proportion change (D) model was trained on a set of n=172 participants, tested on n=38 participants and validated on n=200 participants. Abbreviations: LASSO, Least Absolute Shrinkage and Selection Operator; VAT, Visceral Adipose Tissue; WC, Waist Circumference; MAP, Mean Arterial Pressure; TG, Triglycerides; HDLc, High-Density Lipoprotein cholesterol; GGT, Gamma-Glutamyl Transferase; AST, Aspartate Transaminase; ALKP, alkaline phosphatase; ALT, alanine transaminase; hsCRP, high sensitivity C Reactive Protein; MET, metabolic equivalent for task.


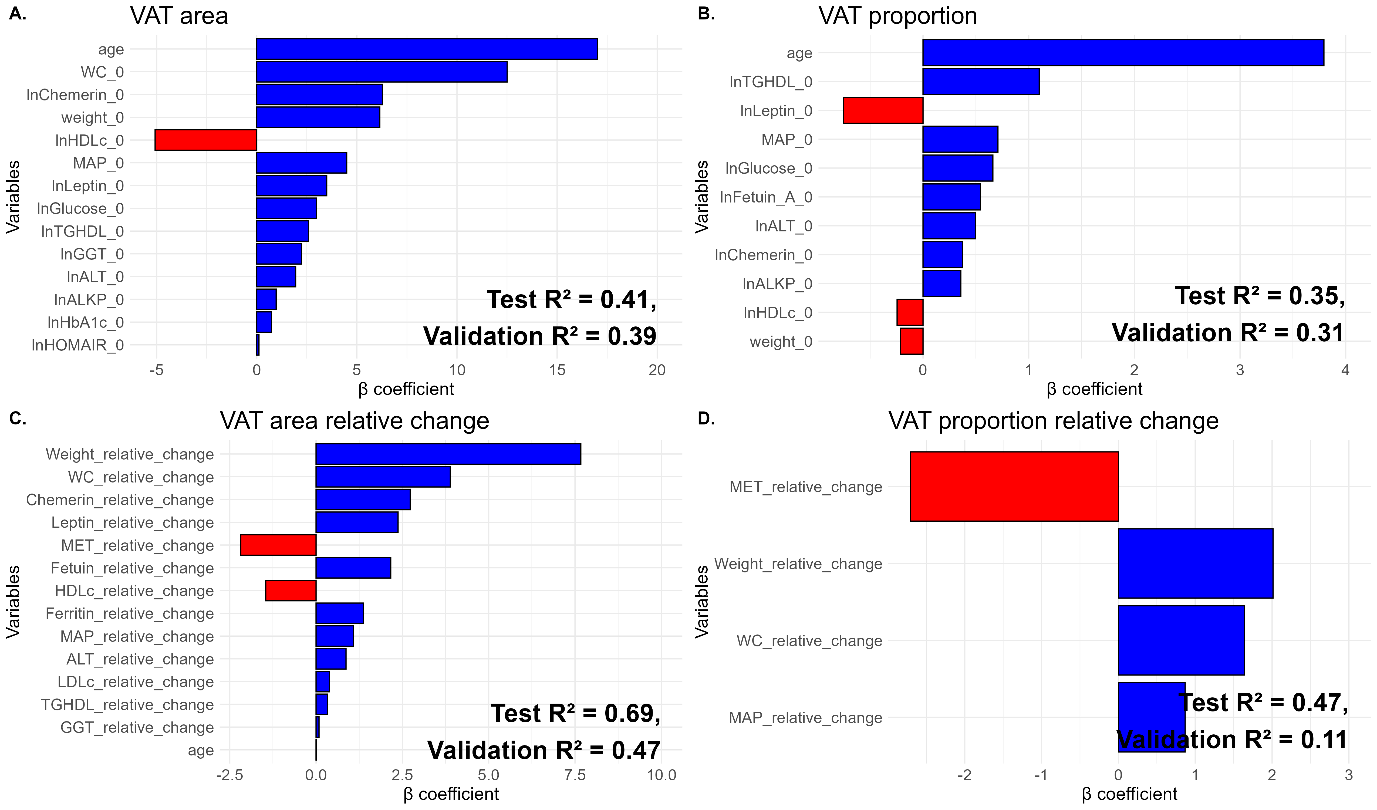


1. ***VAT area*** = 134.65 + 17.012*Age (years) + 12.506*WC (cm) + 6.273*ln(Chemerin) (ng/mL) + 6.146*Weight (kg) - 5.076*ln(HDLc) (mg/dL) + 4.494*MAP (mmHg) + 3.496*ln(Leptin) (ng/mL) + 2.991*ln(Glucose) (mg/dL) + 2.585*ln(TG/HDLc) + 2.23*ln(GGT) (U/L) + 1.945*ln(ALT) (U/L) + 0.97*ln(ALKP) (U/L) + 0.733*ln(HbA1c) (%) + 0.111*ln(HOMA-IR)
2. ***VAT proportion =*** 29.864 + 3.794*Age (years) + 1.1*ln(TG/HDLc) - 0.752*ln(Leptin) (ng/mL) + 0.709*MAP (mmHg) + 0.661*ln(Glucose) (mg/dL) + 0.544*ln(Fetuin-A) (µg/mL) + 0.498*ln(ALT) (U/L) + 0.376*ln(Chemerin) + 0.358*ln(ALKP) (U/L) (ng/mL) - 0.246*ln(HDLc) (mg/dL) - 0.215*Weight (kg)
3. ***VAT area relative change*** = -11.119 + 7.669*Weight_Relative_Change (%) + 3.888*WC_Relative_Change (%) + 2.727*Chemerin_Relative_Change (%) + 2.375*Leptin_Relative_Change (%) - 2.18*MET_Relative_Change (%) + 2.164*Fetuin-A_Relative_Change (%) - 1.457*HDL_Relative_Change (%) + 1.371*Ferritin_Relative_Change (%) + 1.083*MAP_Relative_Change (%) + 0.868*ALT_Relative_Change (%) + 0.392*LDL_Relative_Change (%) + 0.329*TG/HDL_Relative_Change (%) + 0.086*GGT_Relative_Change (%) + 0.005*Age (years)
4. ***VAT proportion relative change*** = -4.081 - 2.71*MET_relative_change (%) + 2.013*Weight_relative_change (%) + 1.642*WC_relative_change (%) + 0.869*MAP_relative_change (%)

**Figure S4. LASSO linear regression models of baseline and change VAT area and proportion while accounting for physical activity (men only).** The x-axis displays the variables selected by the LASSO model and the y-axis represents the estimated β-unstandardized coefficients. The magnitude and direction by which each variable affects baseline VAT area (A) and proportion (B), and changes of VAT area (C) and proportion (D) are represented by the color (blue for positive and red for negative associations) and length of the bars. VAT, visceral adipose tissue. Baseline VAT area (A) model was trained on a set of n=200 participants, tested on n=42 participants and validated on 119 participants. Baseline VAT proportion (B) model was trained on a set of n=192 participants, tested on n=49 participants and validated on n=120 participants. VAT area change (C) model was trained on a set of n=160 participants, tested on n=36 participants and validated on n=94 participants. VAT proportion change (D) model was trained on a set of n=152 participants, tested on n=35 participants and validated on n=183 participants. Abbreviations: LASSO, Least Absolute Shrinkage and Selection Operator; VAT, Visceral Adipose Tissue; WC, Waist Circumference; SBP, Systolic Blood Pressure; DBP, Diastolic Blood Pressure; TG, Triglycerides; HDLc, High-Density Lipoprotein cholesterol; GGT, Gamma-Glutamyl Transferase; AST, Aspartate Transaminase; ALKP, alkaline phosphatase; ALT, alanine transaminase; hsCRP, high sensitivity C Reactive Protein; MET, metabolic equivalent for task.


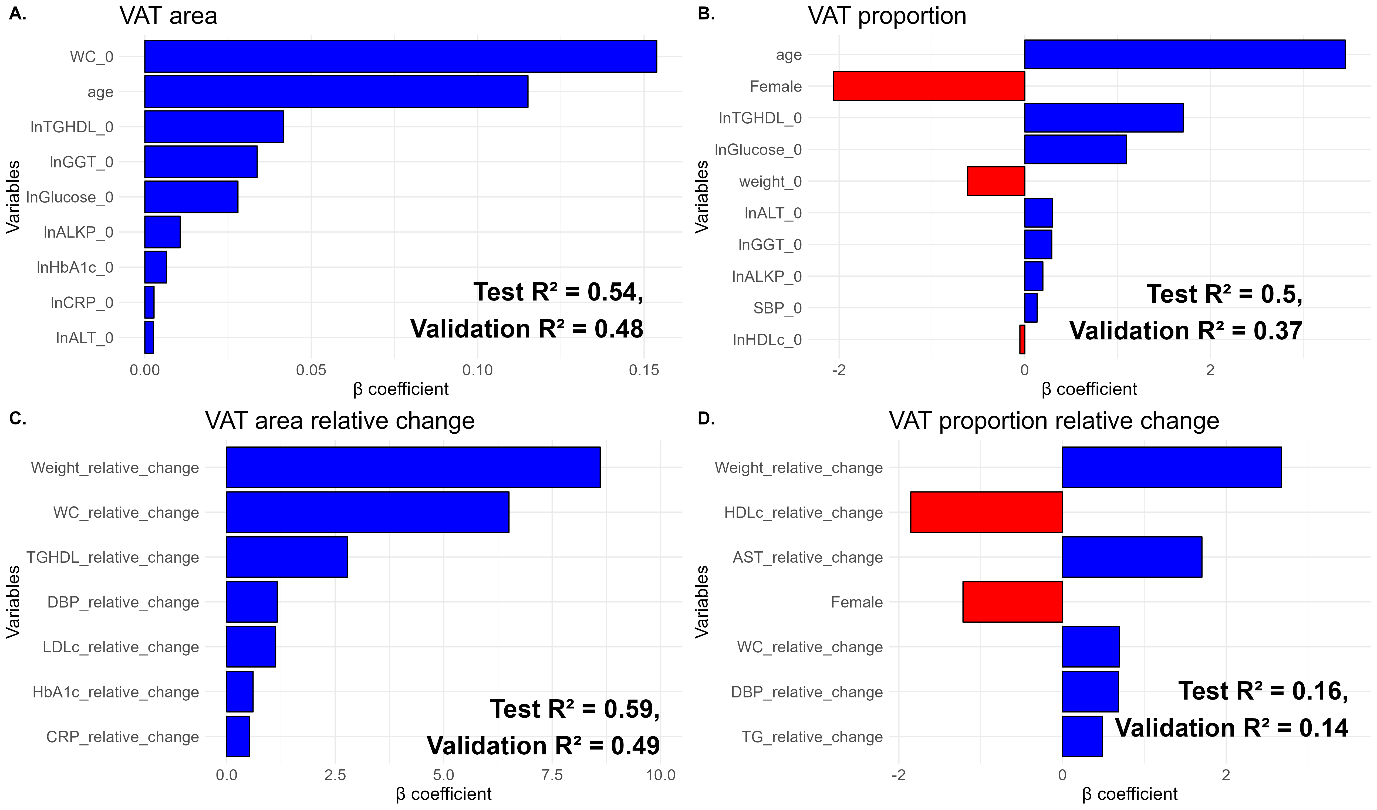


1. ***ln VAT area*** = 4.831 + 0.154*WC (cm) + 0.115*Age (years) + 0.042*ln(TG/HDLc) + 0.034*ln(GGT) (U/L) + 0.028*ln(Glucose) (mg/dL) + 0.011*ln(ALKP) (U/L) + 0.007*ln(HbA1c) (%) + 0.003*ln(hsCRP) (mg/L) + 0.003*ln(ALT) (U/L)
2. ***VAT proportion =*** 29.12 - 0.615*Weight (Kg) + 0.133*SBP (mmHg) + 3.452*Age (years) -2.062*Sex (Male=0, Female=1) + 1.706*ln(TG/HDLc) - 0.053*ln(HDLc) (mg/dL) + 1.096*ln(Glucose) (mg/dL) + 0.29*ln(GGT) (U/L) + 0.195*ln(ALKP) (U/L) + 0.299*ln(ALT) (U/L)
3. ***VAT area relative change*** = -8.852 + 8.607*Weight_relative_change (%) + 6.505*WC_relative_change (%) + 2.786*TG/HDLc_relative_change (%) + 1.164*DBP_relative_change (%) + 1.127*LDLc_relative_change (%) + 0.607*HbA1c_relative_change (%) + 0.52*hsCRP_relative_change (%)
4. ***VAT proportion relative change*** = -3.855 + 2.677*Weight_relative_change (%) -1.854*HDLc_relative_change (%) + 1.704*AST_relative_change (%) -1.216*Sex (Male=0, Female=1) + 0.696*WC_relative_change (%) + 0.684*DBP_relative_change (%) + 0.489*TG_relative_change (%)

**Figure S5. LASSO linear regression models of baseline and change VAT area and proportion for clinical practice application.** The x-axis displays the variables selected by the LASSO model and the y-axis represents the estimated β-unstandardized coefficients. The magnitude and direction by which each variable affects baseline VAT area (A) and proportion (B), and changes of VAT area (C) and proportion (D) are represented by the color (blue for positive and red for negative associations) and length of the bars. VAT, visceral adipose tissue. Baseline VAT area (A) model was trained on a set of n=227 participants, tested on n=57 participants and validated on 146 participants. Baseline VAT proportion (B) model was trained on a set of n=218 participants, tested on n=54 participants and validated on n=146 participants. VAT area change (C) model was trained on a set of n=180 participants, tested on n=42 participants and validated on n=125 participants. VAT proportion change (D) model was trained on a set of n=172 participants, tested on n=42 participants and validated on n=201 participants. Abbreviations: LASSO, Least Absolute Shrinkage and Selection Operator; VAT, Visceral Adipose Tissue; WC, Waist Circumference; SBP, Systolic Blood Pressure; DBP, Diastolic Blood Pressure; TG, Triglycerides; HDLc, High-Density Lipoprotein cholesterol; GGT, Gamma-Glutamyl Transferase; AST, Aspartate Transaminase; ALKP, alkaline phosphatase; ALT, alanine transaminase; hsCRP, high sensitivity C Reactive Protein.

**
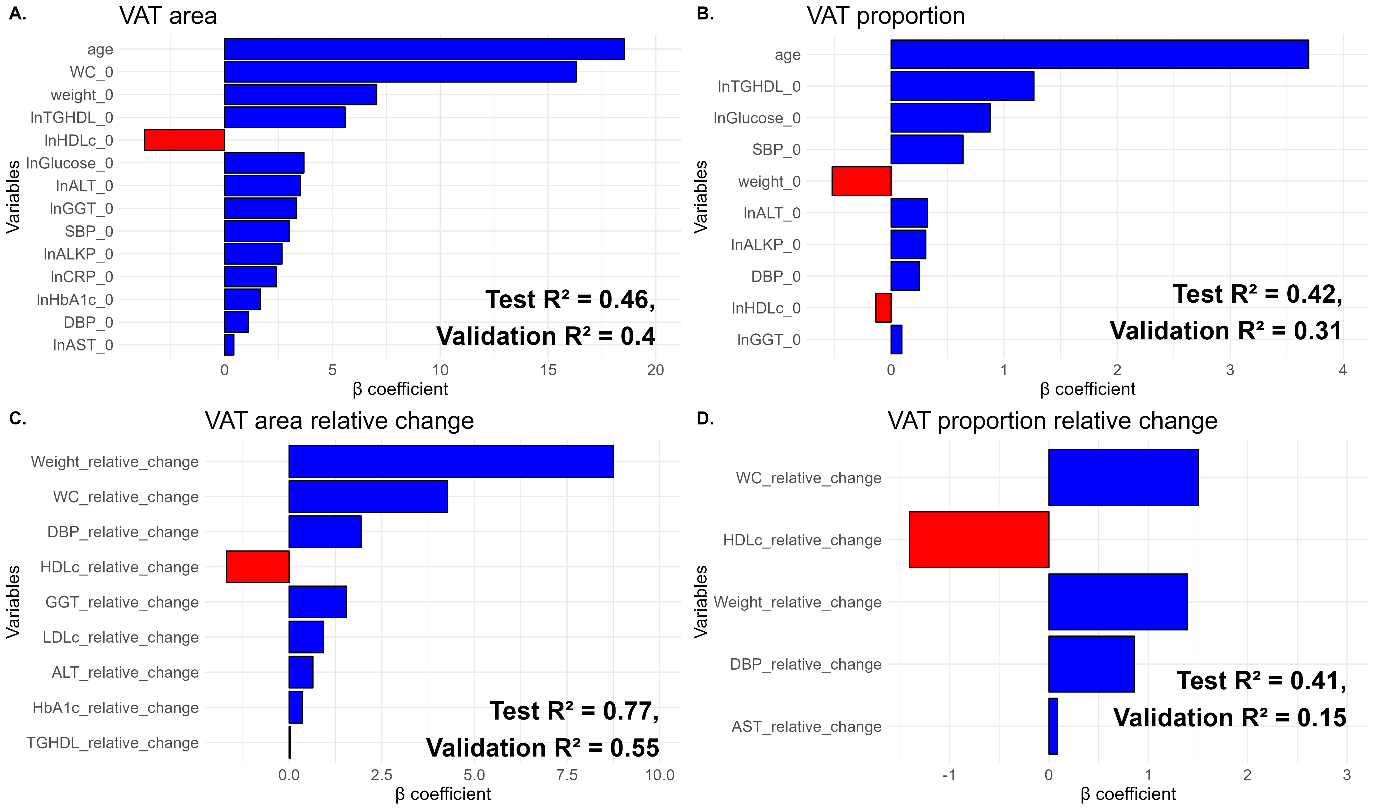
**

1. ***VAT area*** = 135.974 + 18.548*Age (years) + 16.309*WC (cm) + 7.052*Weight (Kg) + 5.592*ln(TG/HDLc) -3.71*ln(HDLc) (mg/dL) + 3.68*ln(Glucose) (mg/dL) + 3.514*ln(ALT) (U/L) + 3.345*ln(GGT) (U/L) + 2.993*SBP (mmHg) + 2.664*ln(ALKP) (U/L) + 2.407*ln(hsCRP) (mg/L) + 1.671*ln(HbA1c) (%) + 1.102*DBP (mmHg) + 0.428*ln(AST) (U/L)
2. ***VAT proportion =*** 30.069 + 3.694*Age (years) + 1.264*ln(TG/HDLc) + 0.876 *ln(Glucose) (mg/dL) + 0.637*SBP (mmHg) - 0.522*Weight (Kg) + 0.323*ln(ALT) (U/L) + 0.307*ln(ALKP) (U/L) + 0.249*DBP (mmHg) - 0.135*ln(HDLc) (mg/dL) + 0.096*ln(GGT) (U/L)
3. ***VAT area relative change*** = -10.161 + 8.753*Weight_relative_change (%) + 4.277*WC_relative_change (%) + 1.944*DBP_relative_change -1.692*HDLc_relative_change (%) + 1.55*GGT_relative_change (%) + 0.931*LDLc_relative_change (%) + 0.642*ALT_relative_change (%) + 0.356*HbA1c_relative_change (%) + 0.028*TG/HDLc_relative_change (%)
4. ***VAT proportion relative change*** = -3.785 + 1.505*WC_relative_change (%) - 1.404*HDLc_relative_change (%) + 1.394*Weight_relative_change (%) + 0.861*DBP_relative_change (%) + 0.085*AST_relative_change (%)

**Figure S6. LASSO linear regression models of baseline and change VAT area and proportion for clinical practice application (men only).** The x-axis displays the variables selected by the LASSO model and the y-axis represents the estimated β-unstandardized coefficients. The magnitude and direction by which each variable affects baseline VAT area (A) and proportion (B), and changes of VAT area (C) and proportion (D) are represented by the color (blue for positive and red for negative associations) and length of the bars. VAT, visceral adipose tissue. Baseline VAT area (A) model was trained on a set of n=200 participants, tested on n=44 participants and validated on 118 participants. Baseline VAT proportion (B) model was trained on a set of n=192 participants, tested on n=46 participants and validated on n=122 participants. VAT area change (C) model was trained on a set of n=160 participants, tested on n=37 participants and validated on n=105 participants. VAT proportion change (D) model was trained on a set of n=152 participants, tested on n=39 participants and validated on n=184 participants. Abbreviations: LASSO, Least Absolute Shrinkage and Selection Operator; VAT, Visceral Adipose Tissue; WC, Waist Circumference; SBP, Systolic Blood Pressure; DBP, Diastolic Blood Pressure; TG, Triglycerides; HDLc, High-Density Lipoprotein cholesterol; GGT, Gamma-Glutamyl Transferase; AST, Aspartate Transaminase; ALKP, alkaline phosphatase; ALT, alanine transaminase; hsCRP, high sensitivity C Reactive Protein.

**References**

1. Thomas EL, Fitzpatrick JA, Malik SJ, Taylor-Robinson SD, Bell JD: **Whole body fat: Content and distribution**. *Progress in Nuclear Magnetic Resonance Spectroscopy* 2013, **73**:56-80.

2. Gepner Y, Shelef I, Schwarzfuchs D, Zelicha H, Tene L, Yaskolka Meir A, Tsaban G, Cohen N, Bril N, Rein M *et al*: **Effect of Distinct Lifestyle Interventions on Mobilization of Fat Storage Pools: CENTRAL Magnetic Resonance Imaging Randomized Controlled Trial**. *Circulation* 2018, **137**(11):1143-1157.

3. Zelicha H, Kloting N, Kaplan A, Yaskolka Meir A, Rinott E, Tsaban G, Chassidim Y, Bluher M, Ceglarek U, Isermann B *et al*: **The effect of high-polyphenol Mediterranean diet on visceral adiposity: the DIRECT PLUS randomized controlled trial**. *BMC Med* 2022, **20**(1):327.

4. Matthews DR, Hosker JP, Rudenski AS, Naylor BA, Treacher DF, Turner RC: **Homeostasis model assessment: insulin resistance and beta-cell function from fasting plasma glucose and insulin concentrations in man**. *Diabetologia* 1985, **28**(7):412-419.
